# Meta-analysis of cognitive functioning in patients with psychotic disorders and obsessive–compulsive symptoms

**DOI:** 10.1007/s00406-020-01174-3

**Published:** 2020-08-11

**Authors:** Lotte Dijkstra, Jentien Vermeulen, Lieuwe de Haan, Frederike Schirmbeck

**Affiliations:** 1 Department of Psychiatry, Amsterdam UMC, University of Amsterdam, Meibergdreef 5, Amsterdam-Zuidoost, 1105 AZ Amsterdam, The Netherlands; 2 Arkin Institute for Mental Health, Amsterdam, The Netherlands

**Keywords:** Neuropsychological, OCD, Schizophrenia, Comorbidity, Neurocognitive

## Abstract

Obsessive–compulsive symptoms (OCS) in psychotic disorders are associated with unfavorable outcomes, whether this extends to cognitive function remains unclear. We conducted meta-analyses on several cognitive domains to investigate overall group differences between patients with a psychotic disorder and co-occurring OCS (OCS +) and those without OCS (OCS−). We used meta-regression to assess possible confounding effects. No overall associations between OCS + and OCS− in any of the 17 investigated cognitive domains were found. We predominantly found large heterogeneity in effect size and direction among studies. Post-hoc analyses of processing speed tasks not purely based on reaction-time showed worse performance in the OCS + group with a small effect size (SMD = − 0.190; *p* = 0.029). Meta-regression revealed advanced age was significantly correlated with worse performance of the OCS + group in processing speed (*R*^2^ = 0.7), working memory (*R*^2^ = 0.11), cognitive inhibition (*R*^2^ = 0.59), and cognitive flexibility (*R*^2^ = 0.34). Patients fulfilling the criteria for an obsessive–compulsive disorder showed less impairment in cognitive inhibition compared to the OCS + group (*R*^2^ = 0.63). Overall, comorbid OCS were not associated with cognitive impairment. However, large heterogeneity between studies highlights the complex nature of factors influencing cognition in people with psychotic disorder and comorbid OCS and warrants further research into possible moderating factors.

**Electronic supplementary material:**

The online version of this article (10.1007/s00406-020-01174-3) contains supplementary material, which is available to authorized users.

## Introduction

Comorbidities, such as depression, substance abuse and anxiety disorders, are highly prevalent in psychotic disorders [1–4]. Amongst common comorbidities are obsessive–compulsive symptoms (OCS) and obsessive–compulsive disorder (OCD). Life-time prevalence of OCS and OCD in people with schizophrenia are, respectively, 31% and 12% [3, 5–8], with symptoms already being present in the at risk mental state (13% and 5%) and during the first episode of psychosis (17% and 7%), but with increasing prevalence in the later stages of disease [3, 9]. There is evidence that patients with comorbid OCS experience more severe psychotic and depressive symptoms, have increased rates of hospitalization and suicide attempts, and experience greater social and occupational impairments compared to schizophrenia patients without OCS [10–12].

In recent years, several reports evaluated the association between OCS and cognitive impairments in patients with psychotic disorders. Cognitive deficits are commonly present in people with schizophrenia as well as in people in an at risk mental state for psychosis [13, 14]. Cognitive deficits have been shown to be one of the most robust predictors of functional outcome in schizophrenia patients [15] and it has been assumed that burden due to comorbid psychopathology such as OCS might increase cognitive deficits in an additive manner. The extent and nature of cognitive deficits in people with psychotic illness and comorbid OCS are not yet clear. Since both schizophrenia and OCD have been consistently linked to impaired executive function, it has been proposed that schizophrenia patients with comorbid OCS might have heightened deficits in this domain [15–19]. In addition, OCD seems to be specifically linked to impairment in cognitive inhibition and flexibility, which are both executive functions [20]. Hence people with psychotic disorder and comorbid OCS might also show additional impairments in these domains. So far, results on executive function in comorbid OCS have been inconsistent. Some studies showed worse performance of comorbid patients in the Wisconsin Card Sorting test or a word fluency test [21–24], whereas others showed better performance in word fluency or the Wisconsin Card Sorting test of the comorbid group [25, 26]. One previous systematic review and meta-analysis by Cunill et al. [27] investigated executive functioning in this population. This meta-analysis demonstrated greater impairment in abstract thinking in the group of patients with OCS compared to the group without OCS, but inconsistent results were found for other executive domains. To the best of our knowledge, no meta-analysis investigated overall group differences between patients with OCS (OCS +) and those without OCS (OCS−) including different cognitive domains such as memory, social cognition, and attention. Furthermore, since the publication of the meta-analysis by Cunill et al. [27], several large studies on this subject have been conducted, allowing for an update of the meta-analytical findings in the executive domain.

### Rationale and aims

The aims of the current study are to [1] examine the association of comorbid OCS in psychotic disorders and performance on different cognitive domains and to [2] examine which patient or study characteristics might explain heterogeneity of result between studies on cognitive function in patients with comorbid OCS. Our hypothesis is that patients with comorbid OCS show heightened cognitive impairment compared to those without comorbid OCS, specifically in the executive domain. Increased knowledge on the type or extent of cognitive dysfunction in patients with comorbid OCS, might have implications for classification and treatment of this patient group.

## Methods

The current meta-analysis was conducted following the guidelines of the PRISMA statement. The study protocol was registered in the PROSPERO database under registration number CRD42019125689.

### Search

The search was performed in cooperation with a clinical librarian (JD). We searched EMBASE, MEDLINE, Web of Science and PsychInfo on 27-5-2019. A set of reference articles and conference abstracts was used to refine the search strategy (supplemental material Sect. 1). In addition, we hand-searched the reference lists of all included articles. The search roughly had three components, namely ‘psychosis’, ‘OCS/OCD’, and ‘cognition’. The full search terms can be found in the supplemental material (Sect. 1).

### Selection criteria and screening

References were screened by two researchers (LD + FS) and added to the initial selection of articles if the title or abstract (1) mentioned obsessive–compulsive symptoms or disorder in a population with psychotic illness, (2) mentioned a cognitive function or cognitive test, (3) mentioned that the study was not a case report, expert opinion, editorial or review, and (4) if the full text was in English, French, Dutch, Spanish, or German. Any discrepancies in selected articles were solved by consensus in a meeting between both researchers. We used the Rayyan app to screen articles and facilitate the comparison of articles between researchers [28]. Studies that were included in the meta-analysis after full-text review had to meet the following criteria: (1) the study evaluated patients with a psychotic illness and determined presence of OCS or OCD, (2) cognitive domains were assessed with neuropsychological tests, and (3) means and standard deviations for the neuropsychological test outcomes were reported in the paper or were made available upon request. Articles were also eligible for inclusion if a sample size and correlations between obsessive–compulsive symptoms and neuropsychological test outcomes were available.

### Data extraction

Data were extracted by one investigator (LD) using a standardized data extraction form, that was developed and tested by two researchers (LD + FS). We emailed authors in case data on neurocognitive test outcomes or confounders were missing from their publication, with at least 3 attempts per publication. The full data extraction form can be accessed in the supplemental material (Sect. 2). In cases, where multiple studies were published by the same author or research group, studies were checked for potential duplicate data. In cases of duplicate data, the largest sample was used. As both cross-sectional and prospective studies were included, only baseline data were extracted from prospective studies.

### Data synthesis and statistical analysis

First, the tests used in each study were classified into a cognitive (sub)domain according to the Strauss and the Lezak compendium of neuropsychological assessments [29, 30]. In addition, we used the classification of the MATRICS Consensus Cognitive Battery initiative, as this battery was specifically designed for people with schizophrenia [31]. Details on how tests were aggregated can be found in the supplemental material (Sect. 3). We subdivided immediate verbal memory into two outcome measures: score of the first trial in a word learning task (‘trial 1 verbal memory’) and sum of immediate verbal memory trials in a word learning task (‘sum of trials verbal memory’), as the second outcome measure contains a learning element and thus measures something distinctly different from the first. In general, meta-analyses were performed in case a minimum of 4 studies were available per domain.

In cases, where authors provided us with full data sets of correlational or unpublished data, we used a YBOCS score of 8 as the cut-off to define the comorbid (OCS +) group, since this was a commonly used cut-off value in the references we included [32–36]. Analyses were restricted to the per study level,for instance, reported subgroups based on disease duration were combined into one OCS + and one OCS− group [37]. In case multiple subgroups based on YBOCS severity were compared, these were combined into one OCS + and one OCS− group. For example, in the study by Ongur et al. [38] we categorized the YBOCS scores up to 11 as the OCS- group and calculated weighted means and SD for this aggregated control group. For the study of Michalopoulou et al. [39] we left out the Stroop task in the cognitive inhibition domain, as it was not clear to us what the values reported in the original study meant.

We used Cohen’s d (standardized mean difference) as the primary measure to evaluate and compare effect sizes. To account for the expected heterogeneity between studies, a random-effects model was used for meta-analyses. The presence of heterogeneity was further evaluated by calculating the *I*^2^ metric.

We examined whether the applied cognitive test within a domain had an effect on the outcome in cases were multiple tests were compiled into one domain, by applying post-hoc sensitivity analysis for each outcome measure that appeared at least 6 times in a domain.

We aimed to perform meta-regression analyses for mean age, gender, mean PANSS positive score and clozapine use in the whole sample. In addition, meta-regression was done for mean YBOCS score of the comorbid group and the categorial variable full diagnoses of OCD vs. presence of OCS as the criterion for the comorbid group. According to methodological guidelines a minimum of ten studies per covariate was assumed appropriate for meta-regression analyses [40].

Comprehensive Meta-analysis software (CMA) version 3 was used for all analyses [41].

### Quality assessment and publication bias

Included studies were assessed for quality using an adapted version of the National Institute of Health (NIH) Quality Assessment Tool for Observational Cohort and Cross-sectional studies by two researchers (LD and FS) and discrepancies were discussed and resolved.

Publication bias was assessed using Rosenthal’s fail safe N and a funnel plot. The above mentioned methods are generally more reliable when applied in a larger number of studies [42, 43]. We did not use statistical tests for funnel plot asymmetry, as these are known to be insensitive in cases, where there are less than ten studies in a meta-analysis [44].

## Results

### Search and screening

**Fig. 1:**
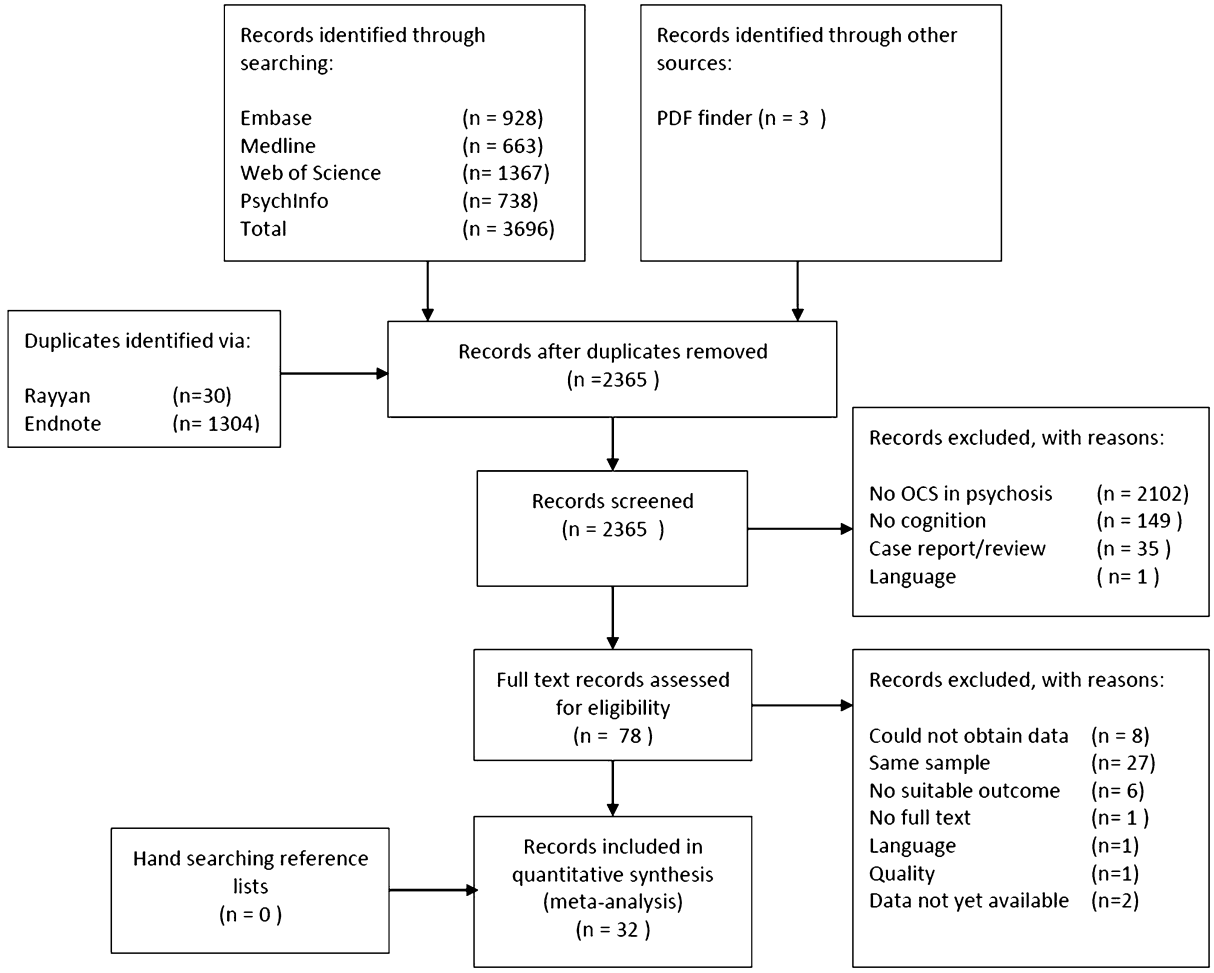
Flow diagram search and screening

The search yielded a total of 2365 references from MEDLINE, EMBASE, PsychInfo, and Web of Science after removing duplicates using Endnote and Rayyan. In addition, we found three publications on the website ‘freefullpdf.com’ that were not indexed in the abovementioned databases [45–47]. Three studies were excluded, because the cognitive test used could not be grouped into a domain and one was excluded for poor quality reporting [47–50]. After full text review, aggregation of cognitive tests, and efforts to obtain data from authors, 32 records were included in the synthesis. Four publications presented data of two overlapping samples, but reporting different outcome measures, so these were combined. The Schulte et al. [51] conference abstract was combined with the corresponding full-text article in the synthesis referred to as ‘Veerman 2016’ [52]. The study of Schirmbeck et al. [33] and Mier et al. [36] were combined and now refer to the ‘Mannheim study’. Finally, access to baseline data of a larger GROUP sample, allowed us to recalculated earlier reported outcomes based on this larger sample [34, 53]. This resulted in a total of 30 studies represented in the meta-analysis. A flow-chart of the search and screening process can be found in Fig. 1.

### Study characteristics

Most studies had a cross-sectional design and included schizophrenia patients in an outpatient setting. Four studies used consecutive sampling and eight used matched sampling, most studies did not report recruitment or sampling strategies. OCS definition was heterogeneous, with a variety of YBOCS cut-off scores or a full DSM diagnosis of OCD. Overall, a total of 2738 patients were included in the presented meta-analyses. Table 1 lists the included studies and their relevant characteristics. Study quality was generally fair and full ratings can be found in the supplemental material (Sect. 4).

**Table 1:** Sample characteristics

References	Study type	Country	Setting	Total N subjects	OCS/OCD definition used	Type of psychosis	Cognitive outcomes reported
Berman [55]	Cross- sectional	US	Inpatient	14 OCS + , 16 OCS−	YBOCS	Schizophrenia	WCST (categories completed), trials A, trials B, delayed visual memory, FAS, block design, similarities, digit symbol coding, digit span *WRAT, information, MMSE*
Bleich-Cohen [56]	Cross- sectional, fMRI	Israel	Inpatient	16 OCS + , 17 OCS−	DSM-IV	Schizophrenia	Nback (accuracy and reaction time)
Borkowska [57]	Cross sectional	Poland	Both in- and outpatient	13 OCS + , 15 OCS−	DSM-IV	Schizophrenia	TMTA, TMTB, Stroop (interference and word reading trial), Verbal fluency (words and perseverations)
Focseneanu [46]	Cross- sectional	Romania	Both in- and outpatient	17 OCS + , 26 OCS−	DSM-IV, obsessions and delusions not related to positive symptoms	Schizophrenia	TMTA, TMTB, Stroop ( word reading, color naming, interference), RAVLT ( sum of tests, *learning rate, curve of doubles, curve of mistakes, volume of recognitions, volume of wrong recognitions*)
Frias [58]	Cross- sectional	Spain	Outpatient	30 OCS + , 37 OCS−	DSM-IV, obsessions and delusions not related to positive symptoms	Schizophrenia and schizoaffective disorder	FAS ( phonemic *and semantic*), block design, digit span, TMTA, TMTB, *working memory subindex IQ score*
GROUP study [34, 53]	Prospective	Netherlands	Both in- and outpatient	145 OCS + , 910 OCS−*	YBOCS at least 8	Non-affective psychosis	WLT (sum of tests and delayed recall), Digit symbol coding, arithmetic, block design, CPT accuracy, RST accuracy, DFAR*, Hinting task, BFRT, information*
Hamid [45]	Cross- sectional	Malaysia	Inpatient	15 OCS + , 85 OCS−	DSM-IV	Schizophrenia	Digit span, RAVLT (trial 1–6 and B list), *MMSE*
Hermesh [59]	Cross- sectional	Israel	Both in- and outpatient	21 OCS + , 19 OCS−	DSM-IV	Schizophrenia	Raven, WCST (categories completed*, errors, perseverations*, perseverative errors, *time per response), alternation learning*
Hwang [21]	Cross- sectional	US	Inpatient	10 OCS + , 10 OCS−	At least three of the operationalized OC symptom criteria for at least 6 months	Schizophrenia	WCST( categories completed, perseverative errors*, non-perseverative errors), MMSE*
Kazhungil [60]	Cross- sectional	India	Inpatient	30 OCS + , 30 OCS−	DSM /SCID I	Schizophrenia	TMTA, TMTB, COWA, Stroop, WCST (perseverative errors and categories completed), *IGT, OAT (total trials and perseverative errors),* color matrix, WMS-LNS, RCFT ( *copy*, immediate recall and delayed recall), RAVLT (*retention,* immediate recall and delayed recall)
Kim [37]	Cross- sectional	Korea	Both in- and outpatient	30 OCS + , 133 OCS−	YBOCS at least 10	Schizophrenia	Digit span test (forward *and backward*), RAVLT ( trial 1, delayed recall *and learning index*), WCST( categories completed, *total errors*), CPT ( reaction time and correct responses), *Finger tapping test*, TMTA, TMTB
Kontis [61]	Cross- sectional	Greece	Inpatient	33 OCS + , 77 OCS−*	YBOCS at least 8	Schizophrenia	CANTAB PRM, *CANTAB SRM*, CANTAB SWM, CANTAB IEDSS, CANTAB SoC
Kumbhani [62]	Cross- sectional	US	Outpatient	29 in total	correlation	Schizophrenia	*WRAT-3*, DKEFS trail making *(visual scanning,* number sequencing*, letter sequencing*, switching, *motor speed*), WCST ( categories completed, perseverative errors, *failure to maintain set)*
Lee [26]	Cross- sectional	Korea	Outpatient	10 OCS + , 17 OCS−	DSM-IV	Schizophrenia	*WAIS IQ, vocabulary,* arithmetic, picture arrangement, block design, information, *picture completion,* RAVLT ( trial 1–5, delayed recall and *delayed recognition*), RCFT ( *copy,* immediate and delayed recall), Stroop (word reading and interference), Fluency, *RFFT*
Lysaker 2000 [35]	Cross- sectional	US	Outpatient	21 OCS + , 25 OCS−	YBOCS at least 8 on either obsession or compulsion	Schizophrenia and schizoaffective disorder	WCST ( categories completed, perseverative errors, *non-perseverative errors, trials to first category*)
Lysaker [16]	Cross- sectional	US	Outpatient	11 OCS + , 52 OCS−	YBOCS at least 17	Schizophrenia and schizoaffective disorder	*WCST (non-perseverative errors*, *other responses*), CPT (hits and false alarms), VRT
Lysaker [22]	Cross- sectional	US	Outpatient	21 OCS + , 45 OCS−	cluster analysis based on YBOCS	Schizophrenia and schizoaffective disorder	WCST (categories completed), CPT (omissions and *commissions)*
Manheim study [33]	Prospective	Germany	Both in- and outpatient	37 OCS + , 43 OCS−	YBOCS at least 8	Schizophrenia and schizoaffective disorder	TMTA, WCST (categories completed and perseveration errors), Stroop, *Go/NoGO, set shifting,* TMTB, N-back, RAVLT ( immediate recall, *interference,* delayed recall), RCFT *( copy* and memory), block design*, d2,* CPT (mistakes), *MWTB*, emotion recognition
Michalopoulou [39]	Cross- sectional	Greece	Outpatient	20 OCS + , 20 OCS−	DSM-IV, obsessions and delusions not related to positive symptoms	Schizophrenia	WCST (Perseverative errors and categories completed), Stroop, COWA, RCFT (*copy* and immediate recall), Block design, Digit span, *Vocabulary*
Ntouros [63]	Cross- sectional	Greece	both in- and outpatient	38 OCS + , 27 OCS−	YBOCS at least 1	Non-affective FEP	Facial affect perception subtest*, CEP, ToM1, ToM2, BEP (happiness, sadness, disgust, surprise, fear, anger, neutral)*
Ongur [61]	Cross- sectional	US	Outpatient	104 OCS−, 14 OCS +	YBOCS at least 11	Schizophrenia and schizoaffective disorder	*CVLT,* TMTB ( seconds and *errors*), Stroop, WCST (perseverative errors and completed categories)
Patel [64]	Cross-sectional	UK	?	12 OCS + , 16 OCS−	DSM-IV	Schizophrenia	ID-ED total errors, SoC problems solved in minimal moves, *CGT (risk taking and decision latency), AGN (total omissions and latency), NART, MCQ-30*
Sahoo [23]	Cross- sectional	India	Both in- and outpatient	40 OCS + , 39 OCS−	DSM-IV	Schizophrenia	TMTA, COWA ( new words*, perseveration words, intrusion words, variant words*), Stroop, TMTB, ToL *( time, moves,* problems solved in minimal moves)
Tiryaki [65]	Cross- sectional	Turkey	Outpatient	22 OCS + , 40 OCS−	DSM-IV, obsessions and delusions not related to positive symptoms	Schizophrenia	TMTA, TMTB, Stroop (reading words*, naming colors* and interference trial), verbal fluency
Tonna [66]	Cross- sectional	Italy	Inpatient	27 OCS + , 34 OCS−*	YBOCS at least 8	Schizophrenia	*MMSE*, MATRICS (processing speed, *attention/vigilance*, working memory, verbal learning, visual learning, problem solving)
Tumkaya [25]	Cross- sectional	Turkey	Outpatient	16 OCS + , 30 OCS−	DSM-IV	Schizophrenia	WCST ( *total errors,* categories completed, *perseverative responses*, perseverative errors, *conceptual responses*), Stroop (word reading, *color naming* and interference), TMT (B-A), WMS-visual memory (immediate and delayed recall), RAVLT ( short term, *learning, inconsistency,* delayed free recall), digit span
Veerman [52, 51]	RCT	Netherlands	Both in- and outpatient	10 OCS + , 39 OCS−*	YBOCS at least 8	Schizophrenia	*MOT (total errors, median latency),* VRM (total correct phase 1*, total correct recognition*), RVP (a, *median latency and probability of hit*), RTI ( *simple RT median,* 5 choice RT median, *error score*), OTS, PAL ( total errors adjusted *and first trial memory score*), SWM *( strategy and* between errors), emotions recognition
Wang [67]	Cross- sectional, fMRI	China	?	22 OCS + , 20 OCS−	DSM-IV	Schizophrenia	*Common sense*, arithmetic, similarities, digit span forward, *digit span backward*
Whitney [32]	Cross- sectional	US	?	26 OCS + , 28 OCS−	YBOCS at least 8 on either obsession or compulsion	Schizophrenia and schizoaffective disorder	*Vocabulary*, WCST (perseverative errors), *BGT (advantageous-disadvantageous)*, RCFT (immediate recall), CVLT, CPT attentiveness score
Whitton [24]	Cross- sectional	Australia	?	34 in total	correlation	Schizophrenia	Ekman 60 faces, *Mind in Eyes, WASI, NART*, HSCT, fluency

*OCS +* people with OCS, *OCS−* people without OCS

? = unclear whether it was an in- or outpatient sample

Sample sizes marked with * indicate these groups were not reported in the original publication, but were calculated from the original data with a YBOCS cut-off of 8

Outcome measures in Italic were not used in the synthesis, since these could not be combined with sufficient other outcomes from other studies to perform meta-analyses

### Quantitative synthesis

**Fig. 2:**
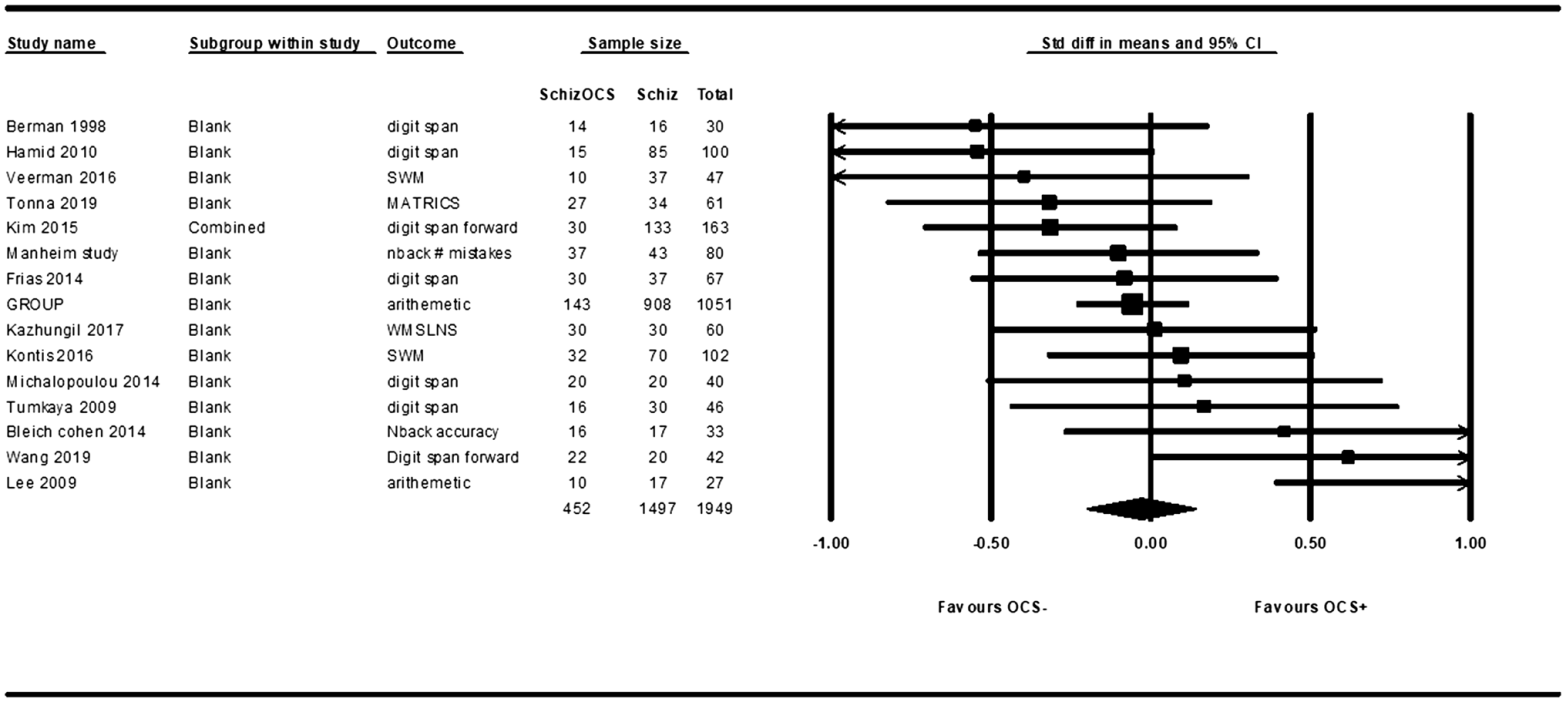
Forest plot working memory. Studies showing better working memory in the OCS− group are plotted on the left and studies showing better working memory the OCS + group are plotted on the right

Meta-analysis was possible for 17 individual domains. As shown in Table 2, meta-analyses were done for attention (with subdomains processing speed and sustained attention), memory (with subdomains working memory, immediate and delayed visual memory, and immediate and delayed verbal memory), executive function (fluency, cognitive inhibition, cognitive flexibility, set-shifting, abstract thinking, planning, and reasoning), facial affect recognition, and visual spatial ability. None of the meta-analyses showed significant results. However, when examining the forest plots, the studies showed a wide spread in effect sizes and even effect directions. Figure 2 shows the condensed forest plot for one of the largest meta-analysis, working memory, as an example of the heterogeneity in effect sizes and directions. Most studies also had a medium to high *I*^2^ statistic [54]. Table 2 shows a summary of the results of the meta-analyses and the corresponding forest plots can be found in the supplemental material (Sect. 5).

**Table 2:** This table shows the results from all meta-analyses

Cognitive domain	*N* studies	*N* patients	SMD	Lower	Upper	*p* Value	*I*^2^
Attention							
Processing speed	17	1946	− 0.133	− 0.300	0.033	0.117	43.009
Sustained attention	7	1457	− 0.107	− 0.271	0.058	0.205	14.176
Memory							
Working memory	15	1949	− 0.030	− 0.201	0.141	0.729	43.787
Immediate visual memory	11	619	− 0.03	− 0.277	0.216	0.810	51.027
Delayed visual memory	4	163	0.051	− 0.263	0.365	0.749	0
Trial 1 verbal memory	6	445	0.224	− 0.195	0.643	0.295	68.432
Sum of trials verbal memory	5	1281	− 0.035	− 0.302	0.232	0.798	50.493
Delayed verbal memory	6	1406	0.023	− 0.115	0.162	0.740	0
Executive function							
Fluency	9	427	− 0.123	− 0.512	0.265	0.534	73.091
Cognitive inhibition	10	576	− 0.208	− 0.489	0.074	0.148	57.745
Cognitive flexibility	12	805	− 0.150	− 0.508	0.208	0.412	80.236
Set shifting	13	1626	− 0.111	− 0.429	0.206	0.492	80.071
Abstract thinking	12	772	− 0.168	− 0.407	0.071	0.169	50.696
Planning	4	250	− 0.229	− 0.802	0.345	0.434	75.815
Reasoning	6	260	− 0.281	− 0.776	0.214	0.265	73.487
Other							
Facial affect recognition	5	1164	− 0.093	− 0.367	0.182	0.507	33.738
Visual spatial ability	6	1304	− 0.038	− 0.352	0.275	0.810	62.005

*N* number, *SMD* standardized mean difference, *Lower* lower limit of confidence interval, *Upper* upper limit of confidence interval

For each meta-analysis the number of studies included, the number of patients those studies represent, the effect measure (standardizes mean difference), the confidence interval, the *p*-value, and the *I*^2^ statistic are shown

**Fig. 3:**
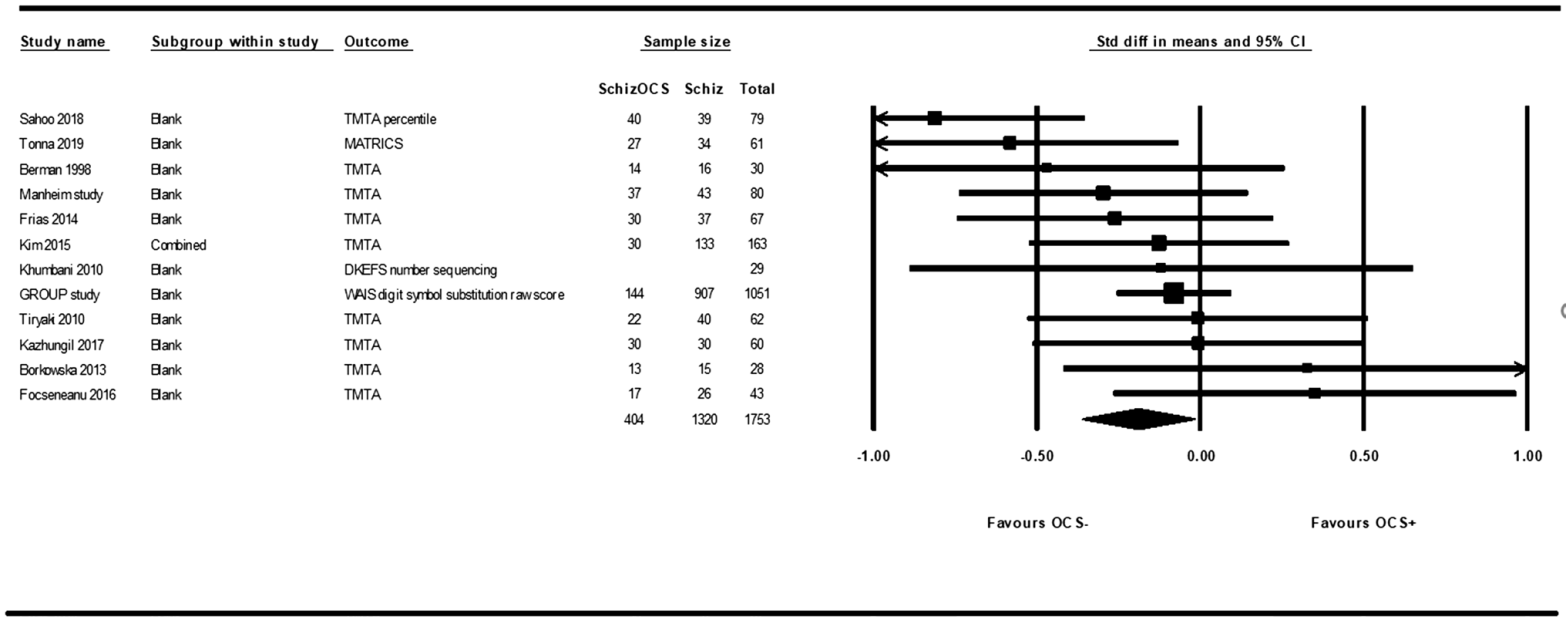
Forest plot processing speed, not purely reaction time based. Studies that showed better reaction time in the OCS− are plotted on the left and studies showing better reaction time in OCS + group are plotted on the right

Post-hoc sensitivity analyses were conducted on more homogenous outcome measures within specific domains (see supplemental material Sect. 5a). Sensitivity analyses on processing speed only including not purely reaction time based outcome measures resulted in significantly worse performance of the OCS + group (SMD = − 0.190, *p* = 0.029) (Fig. 3). No other post-hoc analyses were significant.

### Confounders and meta-regression

Due to a limited number of studies and missing information on confounders in the individual studies, we were only able to conduct meta-regression analyses with the following covariates: YBOCS score for the comorbid group, PANSS positive score for the whole sample, whether a full OCD diagnoses or OCS symptoms were used as the criterion to define the OCS + group, and the mean age for the whole sample. We did not have sufficient studies to examine more than one confounder in a single meta-regression. This resulted in a total of 19 meta-regressions. Meta-regression revealed advanced age was significantly correlated at the *α* = 0.05 level with worse performance of the comorbid group for processing speed (*R*^2^ = 0.7, *p* = 0.029), working memory (*R*^2^ = 0.11, *p* = 0.031), cognitive inhibition (*R*^2^ = 0.59, *p* = 0.024), and cognitive flexibility (*R*^2^ = 0.34, *p* = 0.015). We found that an OCD diagnosis (*n* = 7) instead of OCS (*n* = 3) was associated with better cognitive inhibition of the comorbid group (*R*^2^ = 0.63, *p* = 0.017). Neither PANSS positive score nor severity of OCS (reflected in the mean YBOCS scores) were associated with performance in the cognitive domains in which meta-regression was possible. The results of all meta-regressions can be found in the supplemental material (Sect. 8).

### Publication bias and quality assessment

Publication bias upon visual inspection of the funnel plots was generally low. Rosenthal’s N showed varying degrees of publication bias, but this method is considered to be unreliable for small meta-analyses. Section 7 in the supplemental material shows the publication bias statistics. All included studies were rated for quality and all except one [45] received a rating of ‘fair’ quality. Section 4 in the supplemental material shows the quality assessments for each study.

## Discussion

These comprehensive meta-analyses on the effect of OCS comorbidity on cognition combinedly included 30 studies describing several cognitive domains in a total of 2738 patients. We found no significant associations between functioning in different cognitive domains and the presence of OCS in patients with a psychotic disorder. Only when processing speed was assessed with measures that are not purely reaction time based, we found a small difference (SMD = − 0.190). Our findings do not support the hypothesis that patients with comorbid OCS are more impaired in cognitive functioning, compared to those without OCS. Notably, the heterogeneity in almost all meta-analyses was high, which was evident in the effect directions and effect sizes, as well as the *I*^2^ statistic. We further examined the role of several moderators on cognitive function in patients with comorbid OCS using meta-regression and found that advanced age in the study population was associated with relatively worse performance of the OCS + group in processing speed, working memory, cognitive inhibition, and cognitive flexibility.

Overall, the lack of significant differences in executive functioning stand in contradiction with the findings of the earlier conducted meta-analysis by Cunill et al. [27], which showed impaired abstract thinking in the OCS + group. The current meta-analyses was extended by 13 studies published after 2013 and handled different inclusion criteria, e.g., including correlational data, which resulted in the additional inclusion of three studies published before 2013 [25, 45, 62].

The main finding of our meta-analysis is the large heterogeneity between studies, which reflects the clinical and statistical differences between the individual studies. Without access to primary data, we were unable to fully disentangle this heterogeneity. A possible explanation for the wide dispersion in SMD’s could be that there are two distinct subgroups of people with comorbid OCS; those who have higher cognitive functioning and those who have inferior cognitive functioning compared to patients without OCS. This hypothesis needs further exploration. We attempted to examine factors that might determine these two subgroups using meta-regression. However, due to missing information on relevant variables (such as clozapine use or illness duration) only a limited number of confounders could be investigated. Age seemed to be most clearly associated with cognitive impairment in de OCS + group. Assuming age is a proxy for disease duration, this would be in line with some previous studies that showed OCS might have a protective effect in the early stages of psychotic illness, but a negative effect in the more chronic stage [11, 68–70]. These results might suggest that heterogeneity in our meta-analyses could be explained by differential associations in earlier vs. later disease stages. However, other studies found no moderating effect of illness duration [37].

Notably, studies on first-episode samples only reported better social functioning and less severe negative symptoms in case of co-occurring OCS, but not if criteria for an OCD diagnosis were fulfilled. In line, Meijer et al. [53] noted that in studies reporting an association between worse cognitive functioning and OCS, patients had a relatively high mean YBOCS score. In the current meta-analyses we were not able to demonstrate a possible moderating effect of OCS severity measured with YBOCS total score. However, when investigating a proxy for OCS severity (OCD diagnosis vs. OCS defining the comorbid group), we observed an unexpected result. Contrary to reported higher impairment in cognitive inhibition in primary OCD patients, a comorbid OCD diagnosis in the current meta-regression (and thus more severe symptoms) was associated with less impaired cognitive inhibition. As these findings have not been mentioned previously, careful interpretation and further investigation is warranted. Upon inspection of the underlying studies in this meta-regression, no clear explanation arose for this significant result. Overall, the limited number of possible confounders we were able to investigate, leaves the option open that there are other unknown reasons why some patients with comorbid OCS have improved cognitive outcome, while others have worse cognitive outcome. The use of benzodiazepines and anticholinergic medications for example was often left unassessed, even though they are frequently prescribed in this population, but are also known to impair cognitive performance [71–81]. Several arguments support the assumption that second generation antipsychotics, particularly clozapine, might aggravate or even induce OCS in a subgroup of patients with schizophrenia. In addition, it has been hypothesized that genetic risk-factors might dispose patients to develop these OCS [82]. Unfortunately, neither clozapine use nor genetic information was comprehensively reported. The presence of motor symptoms or extrapyramidal symptoms was also often left unaddressed. Performance on some cognitive tests might be worse because of these symptoms.

Regarding the results of the sensitivity analyses showing more impairment of the OCS group in processing speed, these align with literature in primary OCD. Impaired processing speed has been shown in patients with OCD compared to controls and it has even been hypothesized that processing speed is in fact the primary deficit in OCD [83–85].This could explain why we found a significantly higher impairment in processing speed in psychotic patients with comorbid OCS compared to those without, but not on other domains. However, cautious interpretation is warranted, as the significance of this finding could well be the result of multiple testing. This is strengthened by the fact that we did not demonstrate a significant difference in other cognitive domains that are partly dependent on processing speed, such as cognitive flexibility.

Finally, most of the SMD’s in our study were indeed small, and as people with psychotic illness already have significant cognitive impairments, any additional impairment associated with OCS might be difficult to detect.

### Strengths and limitations

To the best of our knowledge this is the first meta-analysis investigating the association between comorbid OCS and functioning in multiple cognitive domains and the first on this subject to do subsequent meta-regression analyses. Furthermore, detailed search strategies enabled additional inclusions of publications and extensive attempts to obtain unpublished data made it possible to include data that had not been presented in the literature on cognition and OCS in psychotic disorders before.

This meta-analysis has several limitations. Firstly, we combined the results of a variety of neuropsychological tests within domains to increase the power of the meta-analyses, by which we consequently could have introduced heterogeneity within these domains. Over 60 different tests were used in the included studies and in addition, studies sometimes varied in applied scoring systems of these tests. This severely complicates attempts to compare and replicate results. However, where possible we performed subsequent sensitivity analyses with more homogeneous outcome measures (e.g., only using TMTB for cognitive flexibility) that showed comparable results. A second limitation was that some of the meta-analyses only included a small number of studies, which warrants cautious interpretation of the results as meta-analyses with small numbers of studies are less reliable than those with larger numbers of studies. In addition, analyses of multiple outcome measures, performing multiple sensitivity analyses and meta-regressions on the same data might impact the validity of meta-analytical results. As there is an ongoing debate on how to correct for multiplicity, while at the same time being cautious not to decrease power, we did not correct for alpha inflation [86]. However, we acknowledge that statistical significance testing needs cautious interpretation and clinical relevance of the results should rather be interpreted based on the average effects-sizes and confidence intervals. Thirdly, the included studies often only reported a select number of confounders and factors such as depressive symptoms, ethnicity, medication, and education status were often not reported. As mentioned above, some possibly important moderators or confounders which might explain the observed large heterogeneity between studies received very little attention in primary studies. In addition, many included studies used substance (ab)use as an exclusion criterion for selection of participants. This is, therefore, not a likely explanation for the observed heterogeneity in this meta-analysis, but as substance use is highly prevalent in people with psychotic illness [87, 88], current findings are probably not representative of the actual population. Apart from the limitations of the included studies, subgroup analyses and meta-regression can only be applied on the per study level, and factors that might vary between the OCS + and OCS− group or on the per person level, such as for example disease severity or ethnicity, cannot be investigated [40]. Finally, the combination of a small number of studies and missing data on confounders meant that the results of the meta-regression should also be interpreted cautiously. We could only enter one covariate at once due to the small number of studies and we could, therefore, not assess the interaction between covariates such as between age and symptom severity.

### Future directions

In light of our findings, it seems that the way forward for research on cognitive function in people with a psychotic disorder and comorbid OCS is to focus on unveiling the cause for the large heterogeneity in results.

Adopting a dimensional approach could be more suitable to examine the association between cognitive performance and co-occurring OCS. As a variety of factors, among which the severity of OCS, might be at play, creating a dichotomy in people with and without OCS hampers the investigation of OCS severity and other moderating factors. Although some of the studies evaluated the association between dimensional measures [24, 50, 61, 62, 66], most used a categorical approach. Future studies should aim at using methods that allows to capture the complexity of this issue, for which a dimensional approach appears to be the most suitable option available. In this context the YBOCS should be used as the standard instrument as it has been validated in patients with psychotic disorders [89] and would ensure better comparability between individual studies. In addition, more prospective studies should be considered, as they could help shed light not only on the association between comorbid OCS and cognition, but also on the course and nature of the association. As most studies to date have been cross-sectional, causal conclusions cannot be drawn.

Finally, more uniformity should be sought in the type of cognitive tests used in this patient group. A possible solution could be the use of the MATRICS Consensus Cognitive Battery, which was especially developed for people with schizophrenia [31, 90, 91]. This battery has the additional advantage of being relatively fast to administer (65 min), which might make it more suitable for people with more severe psychotic symptoms and which could allow for using it on larger samples. This battery could be supplemented by tasks that test domains that are impaired in primary OCD, such as the Wisconsin Card Sorting Test and the Trail Making Test part B for cognitive inflexibility [20].

## Conclusion

The present meta-analysis highlights the complexity of cognitive function in people with psychotic illness and comorbid OCS. No obvious association between OCS and cognitive function emerges from the analyses, but it raises the question whether perhaps there are distinct groups of people with comorbid OCS; those with better cognitive function and those with worse cognitive function compared to people with psychotic illness without OCS. Our results indicate that age might be a factor that determines those groups, but further research will have to shed light on other factors that might determine cognitive function in patients with comorbid OCS.

## Electronic supplementary material

Below is the link to the electronic supplementary material.

Supplementary file1 (PDF 811 kb)
