# Supplementary material for: Meta-analysis of cognitive functioning in patients with psychotic disorders and obsessive–compulsive symptoms
Source: Eur Arch Psychiatry Clin Neurosci. 2020 Aug 11;271(4):689–706. doi: 10.1007/s00406-020-01174-3 (PMC8119404; doi:10.1007/s00406-020-01174-3)
Supplement: Supplementary file 1 — Supplementary file1 (PDF 811 kb) [file 406_2020_1174_MOESM1_ESM.pdf]

Supplemental material

Meta-analysis of cognitive functioning in patients with psychotic disorders and obsessive-compulsive symptoms.

European Archives of Psychiatry and Clinical Neuroscience. Lotte Dijkstra, Jentien Vermeulen, Lieuwe de Haan,

Frederike Schirmbeck. Amsterdam UMC. Email: n.f.schirmbeck@amsterdamumc.nl.

Table of Contents

1. Reference set of articles and conference abstracts 2
2. Search terms 5
3. Data extraction form 11
4. Result aggregation 12
5. Quality assessment 15
6. Meta-analysis 17
a. Meta-analysis overview table 18
b. Forest plots attention 19
c. Forest plots memory 22
d. Forest plots executive function 29
e. Forest plot facial affect recognition 41
f. Forest plot visual spatial ability 42
7. Confounders per subgroup 43
8. Meta-regression tables 45
9. Publication bias 49
10. Results not included in meta-analysis 50

1. Reference set of articles and conference abstracts

A set of reference articles [1-32] and conference abstracts [33-38] was used to refine search strategy. Below you can find this list.

1. Berman, I., et al., Obsessions and compulsions as a distinct cluster of symptoms in schizophrenia: a neuropsychological study. J Nerv Ment Dis, 1998. 186(3): p. 150-6.

2. Bleich-Cohen, M., et al., Working memory dysfunction in schizophrenia patients with obsessive-compulsive symptoms: an fMRI study. Eur Psychiatry, 2014. 29(3): p. 160-6.

3. Borkowska, A., E. Pilaczynska, and J.K. Rybakowski, The frontal lobe neuropsychological tests in patients with schizophrenia and/or obsessive-compulsive disorder. J Neuropsychiatry Clin Neurosci, 2003. 15(3): p. 359-62.

4. Bozikas, V.P., et al., The role of obsessive-compulsive symptoms in the perception of insincere speech in first-episode psychosis. J Clin Exp Neuropsychol, 2015. 37(8): p. 842-52.

5. Frias, A., et al., Neuropsychological profile and treatment-related features among patients with comorbidity between schizophrenia spectrum disorder and obsessive-compulsive disorder: is there evidence for a "schizo-obsessive" subtype? Psychiatry Res, 2014. 220(3): p. 846-54.

6. Hermesh, H., et al., Alternation learning in OCD/schizophrenia patients. Eur Neuropsychopharmacol, 2003. 13(2): p. 87-91.

7. Hwang, M.Y., J.E. Morgan, and M.F. Losconzcy, Clinical and neuropsychological profiles of obsessive-compulsive schizophrenia: a pilot study. J Neuropsychiatry Clin Neurosci, 2000. 12(1): p. 91-4.

8. Kazhungil, F., et al., Neuropsychological profile of schizophrenia with and without obsessive compulsive disorder. Asian Journal of Psychiatry, 2017. 29: p. 30-34.

9. Kim, S.-W., et al., Associations of obsessive-compulsive symptoms with clinical and neurocognitive features in schizophrenia according to stage of illness. Psychiatry Research, 2015. 226(1): p. 368-75.

10. Kontis, D., et al., Obsessive compulsive symptoms are associated with better functioning independently of cognition in schizophrenia. Comprehensive Psychiatry, 2016. 70: p. 32-40.

11. Kumbhani, S.R., et al., Nonclinical obsessive-compulsive symptoms and executive functions in schizophrenia. J Neuropsychiatry Clin Neurosci, 2010. 22(3): p. 304-12.

12. Lee, M.J., et al., Comparative Analysis of Cognitive Function in Schizophrenia with and without Obsessive Compulsive Disorder. Psychiatry Investig, 2009. 6(4): p. 286-93.

13. Lysaker, P.H., et al., Association of obsessions and compulsions in schizophrenia with neurocognition and negative symptoms. J Neuropsychiatry Clin Neurosci, 2002. 14(4): p. 449-53.

14. Lysaker, P.H., et al., Patterns of obsessive-compulsive symptoms and social function in schizophrenia. Psychiatry Res, 2004. 125(2): p. 139-46.

15. Lysaker, P.H., et al., Obsessive and compulsive symptoms in schizophrenia: clinical and neurocognitive correlates. J Nerv Ment Dis, 2000. 188(2): p. 78-83.

16. Lysaker, P.H., K.A. Whitney, and L.W. Davis, Associations of executive function with concurrent and prospective reports of obsessive-compulsive symptoms in schizophrenia. J Neuropsychiatry Clin Neurosci, 2009. 21(1): p. 38-42.

17. Meijer, J.H., et al., Is a schizo-obsessive subtype associated with cognitive impairment? Results from a large cross-sectional study in patients with psychosis and their unaffected relatives. J Nerv Ment Dis, 2013. 201(1): p. 30-5.

18. Michalopoulou, P.G., et al., Can cognitive deficits differentiate between schizophrenia with and without obsessive–compulsive symptoms? Comprehensive Psychiatry, 2014. 55(4): p. 1015-1021.

19. Ntouros, E., et al., Emotional perception and theory of mind in first episode psychosis: the role of obsessive-compulsive symptomatology. Psychiatry Res, 2014. 220(1-2): p. 112-7.

20. Ongur, D. and D.C. Goff, Obsessive-compulsive symptoms in schizophrenia: associated clinical features, cognitive function and medication status. Schizophr Res, 2005. 75(2-3): p. 349-62.

21. Pallanti, S., et al., Cognitive event-related potentials differentiate schizophrenia with obsessive-compulsive disorder (schizo-OCD) from OCD and schizophrenia without OC symptoms. Psychiatry Res, 2009. 170(1): p. 52-60.

22. Patel, D.D., et al., The neuropsychology of the schizo-obsessive subtype of schizophrenia: a new analysis. Psychol Med, 2010. 40(6): p. 921-33.

23. Sahoo, S., S. Grover, and R. Nehra, Comparison of neurocognitive domains in patients with schizophrenia with and without co-morbid obsessive compulsive disorder. Schizophrenia Research, 2018. 201: p. 151-158.

24. Schirmbeck, F., et al., Stable cognitive deficits in schizophrenia patients with comorbid obsessive-compulsive symptoms: a 12-month longitudinal study. Schizophr Bull, 2013. 39(6): p. 1261-71.

25. Schirmbeck, F., et al., Longitudinal association between cognitive performance and obsessive-compulsive symptoms in patients with psychosis and unaffected siblings. Acta Psychiatr Scand, 2016. 133(5): p. 399-409.

26. Swets, M., et al., Patterns of obsessive-compulsive symptoms and social functioning in schizophrenia; a replication study. Psychiatry Res, 2019. 271: p. 421-427.

27. Tiryaki, A. and E. Ozkorumak, Do the obsessive-compulsive symptoms have an effect in schizophrenia? Compr Psychiatry, 2010. 51(4): p. 357-62.

28. Tonna, M., et al., The "Obsessive Paradox": The Complex Relationship Between Cognitive and Obsessive Dimensions in Schizophrenia. J Nerv Ment Dis, 2019.

29. Tumkaya, S., et al., Schizophrenia with obsessive-compulsive disorder and obsessive-compulsive disorder with poor insight: a neuropsychological comparison. Psychiatry Res, 2009. 165(1-2): p. 38-46.

30. Wehbe, J., et al., Prevalence of Obsessive-Compulsive Disorder in Patients With Schizophrenia and Outcome on Positive and Negative Symptoms, Cognition, and Quality of Life. J Nerv Ment Dis, 2019. 207(4): p. 239-245.

31. Whitney, K.A., et al., Comparative neuropsychological function in obsessive-compulsive disorder and schizophrenia with and without obsessive-compulsive symptoms. Schizophr Res, 2004. 69(1): p. 75-83.

32. Whitton, A.E. and J.D. Henry, The relationship between sub-clinical obsessive-compulsive symptoms and social cognition in chronic schizophrenia. Br J Clin Psychol, 2013. 52(2): p. 115-28.

33. Ongur, D., et al., Obsessive-compulsive symptoms among people with schizophrenia: Relation to psychotic symptoms, cognitive performance and antipsychotic medications. Schizophrenia Research, 2003. 60(1, Supplement): p. 23.

34. Pallanti, S., Anxiety comorbidities and cognitive evoked potential in patients with schizophrenia Schizophrenia Research, 2010. 117(2): p. 210.

35. Theochari, E., et al., Obsessive-compulsive symptoms do not affect visual recognition and working memory in chronic schizophrenia. Schizophrenia Research, 2010. 117(2): p. 324-325.

36. Theochari, E., E. Tsaltas, and D. Kontis, Obsessive compulsive symptoms, social functioning and executive functions in chronic schizophrenia. European Psychiatry, 2017. 41: p. S386.

37. Tumkaya, S., et al., P.1.f.007 Is there neuropsychological support for schizophrenia and obsessive compulsive spectrum disorders. European Neuropsychopharmacology, 2007. 17: p. S300.

38. Bora, L., et al., Prevalence and clinical correlates of comorbid obsessive compulsive disorder in patients with schizophrenia. 2018. 44: p. S362.

2. Search terms

Below you can find the exact search terms used for MEDLINE, EMBASE, PsychINFO, and Web of Science.

	**Ovid MEDLINE(R) ALL 1946 to May 24, 2019**<br>**Search date: 27 May 2019**
**#**	**Searches**
1	"Affective Disorders, Psychotic"/ or "Psychotic Disorders"/ or "Schizophrenia"/ or "Schizophrenia, Catatonic"/ or "Schizophrenia, Disorganized"/ or "Schizophrenia, Paranoid"/
2	(schizophren* or affective psychosis or depressive psychosis or psychotic affective or schizoaffect* or first episode psychosis or schizo-obsessive or schizoobses* or hebephrenia).ab,kf,ti.
3	Brief Psychiatric Rating Scale/
4	(brief psychiatric rating scale or "Scale for the Assessment of Positive Symptoms" or "Scale for the Assessment of Negative Symptoms" or "Positive and Negative Syndrome Scale").ab,kf,ti.
5	((BPRS or SAPS or SANS or PANSS) adj2 (scale? or inventor* or scor* or rating or assessment)).ab,kf,ti.
6	or/1-5
7	Obsessive-Compulsive Disorder/
8	(((ocs or ocd) and obses*) or obsessive compulsive).ab,kf,ti.
9	(yale brown or (maudsley adj3 (inventory or scale))).ab,kf,ti.
10	(("y bocs" or ybocs or oci or moci) adj2 (scale? or inventor* or scor* or rating or assessment)).ab,kf,ti.
11	or/7-10
12	cognitive dysfunction/ or frontal lobe/ or cognition disorders/ or attention/ or executive function/ or psychomotor performance/ or reaction time/ or semantics/ or color perception/ or "discrimination (psychology)"/ or facial expression/ or "inhibition (psychology)"/ or learning/ or verbal learning/ or reversal learning/ or serial learning/ or memory/ or "pattern recognition, visual"/ or problem solving/ or "recognition (psychology)"/ or "retention (psychology)"/ or social perception/ or visual perception/ or wechsler scales/
13	(cogniti* or neurocogniti* or frontal lobe or cognition disorders or attention or executive function or psychomotor performance or reaction time or semantics or color perception or facial expression or inhibition or learning or verbal learning or reversal learning or serial learning or memory or pattern recognition or problem solving or recognition or retention or social perception or visual perception or wechsler scale? or (executive adj3 function*)).ab,kf,ti.

14	(Wisconsin Card Sorting test or WCST or Milwaukee Card Sorting Test or Modified Card Sorting Test or Trail Making Test or TMT or Partington Pathways or Color Trials Test or Stroop test or Stroop task or Color-Word Interference Test or Controlled Oral Word Association Test or COWA or COWAT or Verbal Fluency or Word Fluency or Letter Fluency or FAS or Category Fluency or Phonemic Fluency or Semantic Fluency or Controlled Verbal Fluency or Thurstone Word Fluency Test or Rey-Osterrieth Complex Figure Test or RCFT or ROFT or CFT or Complex Figure Test or Rey Figure or Wechsler Adult Intelligence Scale or WAIS or Wechsler Abbreviated Scale or WASI or Vocabulary or Arithmetic or Digit Span or Letter-Number Sequencing or Picture Completion or Block Design or Matrix Reasoning or Digit Symbol or Symbol Search or Picture Arrangement or Object Assembly or Response shifting Task or RST or Set shifting or Continuous Performance Test or CPT or Cambridge Neuropsychological Test Automated Batteries or CANTAB or Motor Screening or Matching to Sample or Pattern Recognition Memory or Spatial Recognition Memory or Paired Associate Learning or Spatial Span or Spatial Working Memory or Big Little Circle or "Intra/Extradimensional shift" or IED or Rapid Visual Information Processing or Reaction Time or Stockings of Cambridge or National Adult Reading Test or NART or Adult Reading Test or ART or NAART or gambling task or IGT or CVLT or Verbal Learning test or Mini Mental State Examination or MMSE or 3MS or Rey Auditory Verbal Learning Test or RAVLT or AVLT or Finger tapping test or FTT or Finger Oscillation Test or Ruff Figural Fluency Test or RFFT or Benton Visual Retention test or BVRT or Visual retention test or VRT or Benton-test or "Raven's Progressive Matrices" or RPM or Standard Progressive Matrices or SPM or Colored Progressive Matrices or CPM or Alternation learning test or Wechsler Memory Scale or WMS or Hinting Task or Theory of Mind or Wide Range Achievement Test or WRAT or Hopkins Verbal Learning Test or HVLT or Auditory Consonant Trigrams Test or ACTT or Brown-Peterson task or Delis-Kaplan Executive Function System or D-KEFS or DKEFS or Test for Attentional Performance or "Go/NoGo" or Nback or "d2" or Mind in Eyes or "Facial Expression and Emotion Stimuli and Tests" or FEEST or "Ekman 60 faces" or emotion hexagon test or MATRICS or MCCB or Matrics Consensus Cognitive Battery).ab,kf,ti.
15	or/12-14
16	and/6,11,15

	**Ovid Embase Classic+Embase <1947 to 2019 May 24>**<br>**Search date: 27 May 2019**
**#**	**Searches**
1	exp *schizophrenia/ or affective psychosis/ or depressive psychosis/
2	(schizophren* or affective psychosis or depressive psychosis or psychotic affective or schizoaffect* or first episode psychosis or schizo-obsessive or schizoobses* or hebephrenia).ab,kw,ti.
3	Brief Psychiatric Rating Scale/ or "positive and negative syndrome scale"/
4	(brief psychiatric rating scale or "Scale for the Assessment of Positive Symptoms" or "Scale for the Assessment of Negative Symptoms" or "Positive and Negative Syndrome Scale").ab,kw,ti.
5	((BPRS or SAPS or SANS or PANSS) adj2 (scale? or inventor* or scor* or rating or assessment)).ab,kw,ti.

6	or/1-5
7	exp *Obsessive Compulsive Disorder/
8	(((ocs or ocd) and obses*) or obsessive compulsive).ab,kw,ti.
9	Yale Brown Obsessive Compulsive Scale/
10	(yale brown or (maudsley adj3 (inventory or scale))).ab,kw,ti.
11	(("y bocs" or ybocs or oci or moci) adj2 (scale? or inventor* or scor* or rating or assessment)).ab,kw,ti.
12	or/7-11
13	cognitive defect/ or frontal lobe/ or attention/ or executive function/ or psychomotor performance/ or reaction time/ or semantics/ or color vision/ or facial expression/ or learning/ or verbal learning/ or reversal learning/ or sequence learning/ or memory/ or pattern recognition/ or problem solving/ or recognition/ or retention/ or perception/ or wechsler intelligence scale/ or wechsler adult intelligence scale/
14	(cogniti* or neurocogniti* or frontal lobe or cognition disorders or attention or executive function or psychomotor performance or reaction time or semantics or color perception or facial expression or inhibition or learning or verbal learning or reversal learning or serial learning or memory or pattern recognition or problem solving or recognition or retention or social perception or visual perception or wechsler scale? or (executive adj3 function*)).ab,kw,ti.
15	(Wisconsin Card Sorting test or WCST or Milwaukee Card Sorting Test or Modified Card Sorting Test or Trail Making Test or TMT or Partington Pathways or Color Trials Test or Stroop test or Stroop task or Color-Word Interference Test or Controlled Oral Word Association Test or COWA or COWAT or Verbal Fluency or Word Fluency or Letter Fluency or FAS or Category Fluency or Phonemic Fluency or Semantic Fluency or Controlled Verbal Fluency or Thurstone Word Fluency Test or Rey-Osterrieth Complex Figure Test or RCFT or ROFT or CFT or Complex Figure Test or Rey Figure or Wechsler Adult Intelligence Scale or WAIS or Wechsler Abbreviated Scale or WASI or Vocabulary or Arithmetic or Digit Span or Letter-Number Sequencing or Picture Completion or Block Design or Matrix Reasoning or Digit Symbol or Symbol Search or Picture Arrangement or Object Assembly or Response shifting Task or RST or Set shifting or Continuous Performance Test or CPT or Cambridge Neuropsychological Test Automated Batteries or CANTAB or Motor Screening or Matching to Sample or Pattern Recognition Memory or Spatial Recognition Memory or Paired Associate Learning or Spatial Span or Spatial Working Memory or Big Little Circle or "Intra/Extradimensional shift" or IED or Rapid Visual Information Processing or Reaction Time or Stockings of Cambridge or National Adult Reading Test or NART or Adult Reading Test or ART or NAART or gambling task or IGT or CVLT or Verbal Learning test or Mini Mental State Examination or MMSE or 3MS or Rey Auditory Verbal Learning Test or RAVLT or AVLT or Finger tapping test or FTT or Finger Oscillation Test or Ruff Figural Fluency Test or RFFT or Benton Visual Retention test or BVRT or Visual retention test or VRT or Benton-test or "Raven's Progressive Matrices" or RPM or Standard Progressive Matrices or SPM or Colored Progressive Matrices or CPM or Alternation learning test or Wechsler Memory Scale or WMS or Hinting Task or Theory of Mind or Wide Range Achievement Test or WRAT or Hopkins Verbal Learning Test or HVLT or Auditory Consonant Trigrams Test or ACTT or Brown-Peterson task or Delis-Kaplan Executive Function System or D-KEFS or DKEFS or Test for Attentional Performance or "Go/NoGo" or Nback or "d2" or Mind in Eyes or "Facial Expression and Emotion Stimuli and Tests" or FEEST or "Ekman 60 faces" or emotion hexagon test or MATRICS or MCCB or Matrics Consensus Cognitive Battery).ab,kw,ti.

16	or/13-15
17	and/6,12,16

	**Ovid PsycINFO <1806 to May Week 3 2019>**<br>**Search date: 27 May 2019**
**#**	**Searches**
1	exp schizophrenia/ or schizoaffectve disorder/ or affective psychosis/ or "positive and negative symptoms"/
2	(schizophren* or affective psychosis or depressive psychosis or psychotic affective or schizoaffect* or first episode psychosis or schizo-obsessive or schizoobses* or hebephrenia).ab,id,ti.
3	(brief psychiatric rating scale or "Scale for the Assessment of Positive Symptoms" or "Scale for the Assessment of Negative Symptoms" or "Positive and Negative Syndrome Scale").ab,id,ti,tm.
4	((BPRS or SAPS or SANS or PANSS) adj2 (scale? or inventor* or scor* or rating or assessment)).ab,id,ti,tm.
5	or/1-4
6	exp Obsessive Compulsive Disorder/
7	(((ocs or ocd) and obses*) or obsessive compulsive).ab,id,ti.
8	(yale brown or (maudsley adj3 (inventory or scale))).ab,id,ti,tm.
9	(("y bocs" or ybocs or oci or moci) adj2 (scale? or inventor* or scor* or rating or assessment)).ab,id,ti,tm.
10	or/6-9
11	cognitive impairment/ or frontal lobe/ or attention/ or executive function/ or reaction time/ or semantics/ or color perception/ or facial expressions/ or learning/ or verbal learning/ or sequential learning/ or memory/ or "pattern recognition (Cognitive Process)"/ or problem solving/ or "recognition (LEARNING)"/ or retention/ or perception/
12	(cogniti* or neurocogniti* or frontal lobe or cognition disorders or attention or executive function or psychomotor performance or reaction time or semantics or color perception or facial expression or inhibition or learning or verbal learning or reversal learning or serial learning or memory or pattern recognition or problem solving or recognition or retention or social perception or visual perception or wechsler scale? or (executive adj3 function*)).ab,id,ti.

13	(Wisconsin Card Sorting test or WCST or Milwaukee Card Sorting Test or Modified Card Sorting Test or Trail Making Test or TMT or Partington Pathways or Color Trials Test or Stroop test or Stroop task or Color-Word Interference Test or Controlled Oral Word Association Test or COWA or COWAT or Verbal Fluency or Word Fluency or Letter Fluency or FAS or Category Fluency or Phonemic Fluency or Semantic Fluency or Controlled Verbal Fluency or Thurstone Word Fluency Test or Rey-Osterrieth Complex Figure Test or RCFT or ROFT or CFT or Complex Figure Test or Rey Figure or Wechsler Adult Intelligence Scale or WAIS or Wechsler Abbreviated Scale or WASI or Vocabulary or Arithmetic or Digit Span or Letter-Number Sequencing or Picture Completion or Block Design or Matrix Reasoning or Digit Symbol or Symbol Search or Picture Arrangement or Object Assembly or Response shifting Task or RST or Set shifting or Continuous Performance Test or CPT or Cambridge Neuropsychological Test Automated Batteries or CANTAB or Motor Screening or Matching to Sample or Pattern Recognition Memory or Spatial Recognition Memory or Paired Associate Learning or Spatial Span or Spatial Working Memory or Big Little Circle or "Intra/Extradimensional shift" or IED or Rapid Visual Information Processing or Reaction Time or Stockings of Cambridge or National Adult Reading Test or NART or Adult Reading Test or ART or NAART or gambling task or IGT or CVLT or Verbal Learning test or Mini Mental State Examination or MMSE or 3MS or Rey Auditory Verbal Learning Test or RAVLT or AVLT or Finger tapping test or FTT or Finger Oscillation Test or Ruff Figural Fluency Test or RFFT or Benton Visual Retention test or BVRT or Visual retention test or VRT or Benton-test or "Raven's Progressive Matrices" or RPM or Standard Progressive Matrices or SPM or Colored Progressive Matrices or CPM or Alternation learning test or Wechsler Memory Scale or WMS or Hinting Task or Theory of Mind or Wide Range Achievement Test or WRAT or Hopkins Verbal Learning Test or HVLT or Auditory Consonant Trigrams Test or ACTT or Brown-Peterson task or Delis-Kaplan Executive Function System or D-KEFS or DKEFS or Test for Attentional Performance or "Go/NoGo" or Nback or "d2" or Mind in Eyes or "Facial Expression and Emotion Stimuli and Tests" or FEEST or "Ekman 60 faces" or emotion hexagon test or MATRICS or MCCB or Matrics Consensus Cognitive Battery).ab,hw,id,ti,tm.
14	or/11-13
15	and/5,10,14

	**Web of Science Core Collection**<br>**Search date: 27 May 2019**
**#**	**Searches**
1	TS=("positive and negative symptoms" OR schizophren* or "affective psychosis" or "depressive psychosis" or "psychotic affective" or schizoaffect* or "first episode psychosis" or "schizo-obsessive" or schizoobses* or hebephrenia)
2	TS=("brief psychiatric rating scale" or "Scale for the Assessment of Positive Symptoms" or "Scale for the Assessment of Negative Symptoms" or "Positive and Negative Syndrome Scale")
3	TS=((BPRS or SAPS or SANS or PANSS) NEAR/1 (scale? or inventor* or scor* or rating or assessment))
4	#1 OR #2 OR #3
5	TS=("obsessive compulsive")

6	TS=("yale brown" or (maudsley NEAR/2 (inventory or scale)))
7	TS=(("y bocs" or ybocs or oci or moci) NEAR/1 (scale? or inventor* or scor* or rating or assessment))
8	#5 OR #6 OR #7
9	TS=(cogniti* or neurocogniti* or frontal lobe or cognition disorders or attention or executive function or psychomotor performance or reaction time or semantics or color perception or facial expression or inhibition or learning or verbal learning or reversal learning or serial learning or memory or pattern recognition or problem solving or recognition or retention or social perception or visual perception or wechsler scale? or (executive NEAR/2 function*))
10	TS=(Wisconsin Card Sorting test or WCST or Milwaukee Card Sorting Test or Modified Card Sorting Test or Trail Making Test or TMT or Partington Pathways or Color Trials Test or Stroop test or Stroop task or Color-Word Interference Test or Controlled Oral Word Association Test or COWA or COWAT or Verbal Fluency or Word Fluency or Letter Fluency or FAS or Category Fluency or Phonemic Fluency or Semantic Fluency or Controlled Verbal Fluency or Thurstone Word Fluency Test or Rey-Osterrieth Complex Figure Test or RCFT or ROFT or CFT or Complex Figure Test or Rey Figure or Wechsler Adult Intelligence Scale or WAIS or Wechsler Abbreviated Scale or WASI or Vocabulary or Arithmetic or Digit Span or Letter-Number Sequencing or Picture Completion or Block Design or Matrix Reasoning or Digit Symbol or Symbol Search or Picture Arrangement or Object Assembly or Response shifting Task or RST or Set shifting or Continuous Performance Test or CPT or Cambridge Neuropsychological Test Automated Batteries or CANTAB or Motor Screening or Matching to Sample or Pattern Recognition Memory or Spatial Recognition Memory or Paired Associate Learning or Spatial Span or Spatial Working Memory or Big Little Circle or "Intra/Extradimensional shift" or IED or Rapid Visual Information Processing or Reaction Time or Stockings of Cambridge or National Adult Reading Test or NART or Adult Reading Test or ART or NAART or gambling task or IGT or CVLT or Verbal Learning test or Mini Mental State Examination or MMSE or 3MS or Rey Auditory Verbal Learning Test or RAVLT or AVLT or Finger tapping test or FTT or Finger Oscillation Test or Ruff Figural Fluency Test or RFFT or Benton Visual Retention test or BVRT or Visual retention test or VRT or Benton-test or "Raven's Progressive Matrices" or RPM or Standard Progressive Matrices or SPM or Colored Progressive Matrices or CPM or Alternation learning test or Wechsler Memory Scale or WMS or Hinting Task or Theory of Mind or Wide Range Achievement Test or WRAT or Hopkins Verbal Learning Test or HVLT or Auditory Consonant Trigrams Test or ACTT or Brown-Peterson task or Delis-Kaplan Executive Function System or D-KEFS or DKEFS or Test for Attentional Performance or "Go/NoGo" or Nback or "d2" or Mind in Eyes or "Facial Expression and Emotion Stimuli and Tests" or FEEST or "Ekman 60 faces" or emotion hexagon test or MATRICS or MCCB or Matrics Consensus Cognitive Battery)
11	#9 OR #10
12	#4 AND #8 AND #11

3. Data extraction form

This is the list of items in the data-extraction form. The original data extraction form was an excel sheet, but it is represented as a list for the purpose of readability.

Per study data

1. Author
2. Year of publication
3. Title of publication
4. DOI number
5. Study type
   (e.g. prospective, cross-sectional, RCT)
6. Country of study
7. Setting
   (e.g. in- or outpatient)
8. Number of study participants
9. Mean age
10. % Male
11. Ethnicity
    % of ethnic groups
12. First episode sample?
13. Instrument used to assess OCS
14. How OCS/OCD was defined
15. Type of psychosis
    (e.g. non-affective, first episode, schizophrenia only)
16. Instrument used to assess psychotic illness
17. PANSS total score
18. PANSS positive score
19. PANSS negative score
20. PANSS GPP score
21. Antipsychotic medication
22. Substance abuse as exclusion criterion?
23. Instruments used to assess other comorbidities
    (e.g. HDRS)
24. Cognitive tests reported
25. Corrected for confounders?
26. Cognitive domain according to authors
27. Authors own conclusions

Cognitive test outcomes

28. N per subgroup
29. Mean + SD for test outcomes
30. Effect sizes
31. Correlation coefficient
32. P-value

Confounders per OCS+ or OCS- subgroup

33. Age (Mean+ SD)
34. % Male
35. Duration of psychotic illness (Mean+ SD)
36. Age of onset (Mean+ SD)
37. PANSS total (Mean+ SD)
38. PANSS positive (Mean+ SD)
39. PANSS negative (Mean+ SD)
40. PANSS GPP (Mean+ SD)
41. Antipsychotic medication
    % per drug
42. Clozapine use %
43. 2nd generation antipsychotic use %
44. Antidepressant use %
45. Other comorbidity
    % of other comorbidities if reported
46. YBOCS score
    (Mean+ SD)
47. Education in years (Mean+ SD)

4. Result aggregation

When classifying tests and their respective test measures into cognitive domains, we aimed to create categories that were as homogenous as possible, while simultaneously avoiding making too many categories with a small number of studies barring the possibility to conduct a meta-analysis. We determined a minimum of four studies within a category were required to perform meta-analysis.

Each of the following tables shows which cognitive tests and corresponding measures within that test were combined into a domain. The final table shows an overview of measures that were reported in insufficient studies to be combined into a meta-analysis. Note that the order of presentation in each table is also the order of preference in the analysis. E.g. if a study reported both TMT A and digit symbol coding, TMT A was used for the analysis.

Visual spatial abilities	Test measure
block design	-

Social cognition:	Test measure
**Facial affect recognition**	
DFAR	-
Ekman 60 faces	-
PESIT	Facial affect perception
Cantab emotion recognition	-
Emotion recognition	-

Attention	Test measure
**Processing speed not RT based:**	
TMT A	seconds
Digit Symbol Coding	-
MATRICS	-
DKEFS number sequencing	-
**Processing speed RT based:**	
CPT	RT
Nback	RT
Cantab RTI	5 choice RT
Stroop word reading	seconds
**Sustained attention:**	
CPT	Accuracy/omission/hits
Cantab RVP	A

Memory	Test measure
**Immediate visual memory**	
RCFT	Recall trial/ immediate
MATRICS (BVMT-R)	Total recall over 3 trials
WMS visual memory	immediate
VRT	-
‘immediate visual memory’	-
Cantab PAL	Total errors adjusted
Cantab PRM	% correct
**Delayed visual memory**	
RCFT	delayed
‘delayed visual memory’	-
WMS visual memory	Delayed recall
**Working memory:**	
digit span	(forward)
arithmetic	-
Letter Number Sequencing	-
Nback	Accuracy/mistakes
SWM	errors
MATRICS	-
**Trial 1 verbal memory***	
RAVLT	A1/trial 1/immediate recall/short term memory
Cantab VRM	Total correct phase 1

Memory continued	Test measure
**Sum of tests verbal learning***	
RAVLT	Sum of tests
WLT	Immediate recall
CVLT	-
MATRICS	-
**Delayed verbal memory/ word retention**	
RAVLT	Delayed recall
WLT	Retention rate

* Immediate verbal memory was split into two categories as some studies reported the score of the first trial of a word learning task (trial 1 verbal memory), while others reported the sum of all immediate verbal memory trials (sum of tests verbal memory). As the second has a learning component to it and the first does not, these two were thought to measure something different and were thus taken separately. For example, the RAVLT has 5 immediate verbal learning trials and the first trial was put in the ‘trial 1’ category and the sum of the 5 trials into ‘sum of trials’.

Executive Function	Test measure
**Set shifting:**	
WCST	Perseverative errors
RST	accuracy
IED	Total errors (adjusted)
**Abstract thinking**	
WCST	Categories completed
**Cognitive flexibility:**	
TMT B (or B-A)	Seconds or percentile
DKEFS trail making switching	-
**Cognitive inhibition:**	
Stroop interference	-
Hayling Sentence Completion	-
Go/NoGo	-
**Planning:**	
SoC	Problems solved in min. moves
ToL 3	Problems solved in min. moves
Cantab OTS	Problems solved in first choice
**Verbal Fluency:**	
COWA/FAS	N word (phonemic)
**Reasoning/problem solving**	
WAIS picture arrangement	-
MATRICS mazes	-
Raven	-
WAIS picture completion	-
WAIS color matrix	-
WAIS similarities	-

Insufficient studies	N studies
gambling (CGT/IGT/BGT)	3
Affective GoNoGo	1
DRT *	1
alternation learning	1
OAT	1
RFFT	1
d2	1
Cantab SRM	1
MMSE	3
WAIS information	4
WAIS vocabulary	3
NART	2
WRAT	2
WAIS IQ	2
WASI	1
MWTB	1
Hinting task	1
ToM	1
Mind in Eyes	1
BFRT	1

5. Quality assessment

Below you can find the questions from the National Institute of Health Quality Assessment Tool for Observational Cohort and Cross-Sectional Studies. Note that we did not use the question marked in grey, as they apply to prospective studies and we only included cross sectional data in our analysis.

1. Was the research question or objective in this paper clearly stated?
2. Was the study population clearly specified and defined?
3. Was the participation rate of eligible persons at least 50%?
4. Were all the subjects selected or recruited from the same or similar populations (including the same time period)? Were inclusion and exclusion criteria for being in the study pre-specified and applied uniformly to all participants?
5. Was a sample size justification, power description, or variance and effect estimates provided?
6. For the analyses in this paper, were the exposure(s) of interest measured prior to the outcome(s) being measured?
7. Was the timeframe sufficient so that one could reasonably expect to see an association between exposure and outcome if it existed?
8. For exposures that can vary in amount or level, did the study examine different levels of the exposure as related to the outcome (e.g., categories of exposure, or exposure measured as continuous variable)?
9. Were the exposure measures (independent variables) clearly defined, valid, reliable, and implemented consistently across all study participants?
10. Was the exposure(s) assessed more than once over time?
11. Were the outcome measures (dependent variables) clearly defined, valid, reliable, and implemented consistently across all study participants?
12. Were the outcome assessors blinded to the exposure status of participants?
13. Was loss to follow-up after baseline 20% or less?
14. Were key potential confounding variables measured and adjusted statistically for their impact on the relationship between exposure(s) and outcome(s)?

On the next page you will find the detailed quality assessment per question for each study.

Author& Year	1	2	3	4	5	6	8	9	11	12	14	Rating	Motivation
Berman 1998	Yes	Yes	NR	CD	No	No	Yes	Yes	Yes	Yes	No	Fair	
Bleich-Cohen 2014	Yes	Yes	NR	Yes	No	No	No	Yes	Yes	CD	No	Fair	
Borkowska 2003	Yes	Yes	NR	CD	No	No	No	CD	Yes	CD	No	Fair	
Focseneanu 2016	Yes	Yes	NR	CD	No	No	No	No	Yes	CD	Yes	Fair	
Frias 2014	Yes	Yes	Yes	Yes	Yes	No	No	CD	Yes	CD	Yes	Fair	
GROUP study	Yes	Yes	NR	CD	No	No	No	Yes	Yes	CD	Yes	Fair	
Hamid 2010	Yes	Yes	NR	Yes	No	No	No	Yes	No	No	No	Poor	Many things not reported
Hermesh 2003	Yes	Yes	NR	CD	No	No	Yes	Yes	Yes	Yes	No	Fair	
Hwang 2000	Yes	Yes	NR	CD	No	No	No	Yes	Yes	Yes	No	Fair	
Kazhungil 2017	Yes	Yes	No	Yes	No	No	No	CD	Yes	No	Yes	Fair	
Kim 2015	Yes	Yes	CD	Yes	No	No	No	Yes	Yes	Yes	Yes	Fair	
Kontis 2016	Yes	Yes	Yes	Yes	Yes	No	Yes	Yes	Yes	CD	Yes	Fair	
Kumbhani 2010	Yes	Yes	NR	CD	Yes	No	Yes	Yes	Yes	CD	Yes	Fair	
Lee 2009	Yes	Yes	No	Yes	No	No	Yes	Yes	Yes	Yes	Yes	Fair	
Lysaker 2000	Yes	Yes	NR	CD	Yes	No	No	Yes	Yes	No	Yes	Fair	
Lysaker 2002	Yes	Yes	NR	CD	No	No	No	Yes	Yes	CD	Yes	Fair	
Lysaker 2004	Yes	Yes	NR	CD	No	No	Yes	Yes	Yes	Yes	Yes	Fair	
Manheim study	Yes	Yes	Yes	CD	Yes	CD	Yes	Yes	Yes	CD	Yes	Fair	
Meijer 2013	Yes	Yes	NR	CD	Yes	No	Yes	Yes	Yes	CD	Yes	Fair	
Michalopoulou 2014	Yes	Yes	NR	CD	No	No	Yes	No	Yes	CD	Yes	Fair	
Ntouros 2014	Yes	Yes	NR	CD	Yes	No	No	Yes	Yes	CD	Yes	Fair	
Ongur 2005	Yes	Yes	NR	Yes	No	No	Yes	Yes	Yes	CD	Yes	Fair	
Patel 2010	Yes	Yes	NR	CD	No	No	Yes	Yes	Yes	CD	No	Fair	
Sahoo 2018	Yes	Yes	NR	CD	Yes	No	Yes	Yes	Yes	CD	Yes	Fair	
Tiryaki 2010	Yes	Yes	NR	CD	No	No	Yes	Yes	Yes	No	No	Fair	
Tonna 2019	Yes	Yes	NR	Yes	Yes	No	Yes	Yes	Yes	CD	No	Fair	
Tumkaya 2009	Yes	Yes	NR	Yes	Yes	No	No	Yes	Yes	Yes	No	Fair	
Veerman 2016	Yes	Yes	No	Yes	Yes	Yes	No	Yes	Yes	Yes	Yes	Fair	
Wang 2019	Yes	Yes	NR	CD	No	Yes	No	No	Yes	CD	Yes	Fair	
Whitney 2003	Yes	Yes	NR	CD	Yes	No	Yes	Yes	Yes	Yes	Yes	Fair	
Whitton 2013	Yes	Yes	NR	CD	Yes	No	Yes	Yes	Yes	CD	Yes	Fair	

Quality assessment for each study. CD=cannot determine and NR=not reported

6. Meta-analysis

In the following pages you can find an overview table of the meta-analysis including the post-hoc sensitivity analyses and the forest plots for each individual meta-analysis. Note that in all cases studies where patients without obsessive-compulsive symptoms performed better are plotted on the left and that studies where the patients with obsessive-compulsive symptoms performed better are plotted on the right. All studies are sorted by SMD from low to high in the forest plot.

a. Meta-analysis overview table

Cognitive domain	N studies	N patients	SMD	Lower	Upper	p-value	I^2
**Attention**							
Processing speed	17	1946	-0.133	-0.300	0.033	0.117	43.009
**Processing speed: TMTA*	9	612	-0.177	-0.406	0.052	0.129	42.069
***Processing speed: not purely RT based*	12	1753	**-0.190**	**-0.362**	**-0.019**	**0.029**	36.574
***Processing speed: purely RT based*	9	489	-0.105	-0.413	0.203	0.504	55.279
Sustained attention	7	1457	-0.107	-0.271	0.058	0.205	14.176
**Memory**							
Working memory	15	1949	-0.030	-0.201	0.141	0.729	43.787
**Working memory: digit span*	7	488	-0.097	-0.386	0.192	0.511	46.907
Immediate visual memory	11	619	-0.03	-0.277	0.216	0.810	51.027
Delayed visual memory	4	163	0.051	-0.263	0.365	0.749	0
Trial 1 verbal memory	6	445	0.224	-0.195	0.643	0.295	68.432
Sum of trials verbal memory	5	1281	-0.035	-0.302	0.232	0.798	50.493
Delayed verbal memory	6	1406	0.023	-0.115	0.162	0.740	0
**Executive function**							
Fluency	9	427	-0.123	-0.512	0.265	0.534	73.091
Cognitive inhibition	10	576	-0.208	-0.489	0.074	0.148	57.745
**Cognitive inhibition: Stroop*	9	542	-0.208	-0.516	0.100	0.186	62.427
Cognitive flexibility	12	805	-0.150	-0.508	0.208	0.412	80.236
**Cognitive flexibility: TMTB seconds*	9	651	-0.152	-0.575	0.271	0.482	81.811
Set shifting	13	1626	-0.111	-0.429	0.206	0.492	80.071
**Set shifting: WCST*	10	596	-0.081	-0.549	0.387	0.735	83.392
Abstract thinking	12	772	-0.168	-0.407	0.071	0.169	50.696
Planning	4	250	-0.229	-0.802	0.345	0.434	75.815
Reasoning	6	260	-0.281	-0.776	0.214	0.265	73.487
**Other**							
Facial affect recognition	5	1164	-0.093	-0.367	0.182	0.507	33.738
Visual spatial ability	6	1304	-0.038	-0.352	0.275	0.810	62.005

*Results from meta-analyses. Results marked with * are subgroup analyses on specific cognitive tests done as sensitivity analyses. Results marked with ** are post-hoc subgroup analyses. N= number, SMD = standardized mean difference, Lower = lower limit of confidence interval, Upper = upper limit of confidence interval.*

b. Forest plots attention

Study name	Subgroup within study	Outcome	Statistics for each study							Std diff in means and 95% CI
			Std diff in means	Standard error	Variance	Lower limit	Upper limit	Z-Value	p-Value	
Sahoo 2018	Blank	TMTA percentile	-0.812	0.234	0.055	-1.271	-0.353	-3.469	0.001	
Tonna 2019	Blank	MATRICS	-0.582	0.263	0.069	-1.098	-0.066	-2.212	0.027	
Berman 1998	Blank	TMTA	-0.470	0.371	0.138	-1.197	0.257	-1.266	0.205	
Lee 2009	Blank	Stroop simple	-0.333	0.401	0.161	-1.119	0.454	-0.829	0.407	
Manheim study	Blank	TMTA	-0.297	0.225	0.051	-0.738	0.145	-1.315	0.188	
Frias 2014	Blank	TMTA	-0.262	0.247	0.061	-0.745	0.222	-1.060	0.289	
Tumkaya 2009	Blank	Stroop word	-0.227	0.310	0.096	-0.835	0.382	-0.730	0.465	
Bleich Cohen	Blank	Nback RT	-0.133	0.349	0.122	-0.817	0.550	-0.382	0.703	
Kim 2015	Combined	TMTA	-0.126	0.202	0.041	-0.523	0.270	-0.625	0.532	
Khumbani 2010	Blank	DKEFS number sequencing	-0.120	0.393	0.154	-0.890	0.650	-0.306	0.760	
GROUP study	Blank	WAIS digit symbol substitution raw score	-0.081	0.090	0.008	-0.256	0.095	-0.898	0.369	
Tiryaki 2010	Blank	TMTA	-0.008	0.265	0.070	-0.529	0.512	-0.032	0.975	
Kazhungil 2017	Blank	TMTA	-0.006	0.258	0.067	-0.512	0.500	-0.024	0.981	
Veerman 2016	Blank	RTI 5 choice RT	0.077	0.356	0.127	-0.622	0.776	0.216	0.829	
Borkowska 2013	Blank	TMTA	0.327	0.381	0.146	-0.420	1.075	0.858	0.391	
Focseneanu 2016	Blank	TMTA	0.350	0.314	0.099	-0.265	0.966	1.116	0.265	
Michalopoulou 2014	Blank	Stroop word	0.907	0.332	0.110	0.256	1.558	2.731	0.006	
			-0.133	0.085	0.007	-0.300	0.033	-1.569	0.117	

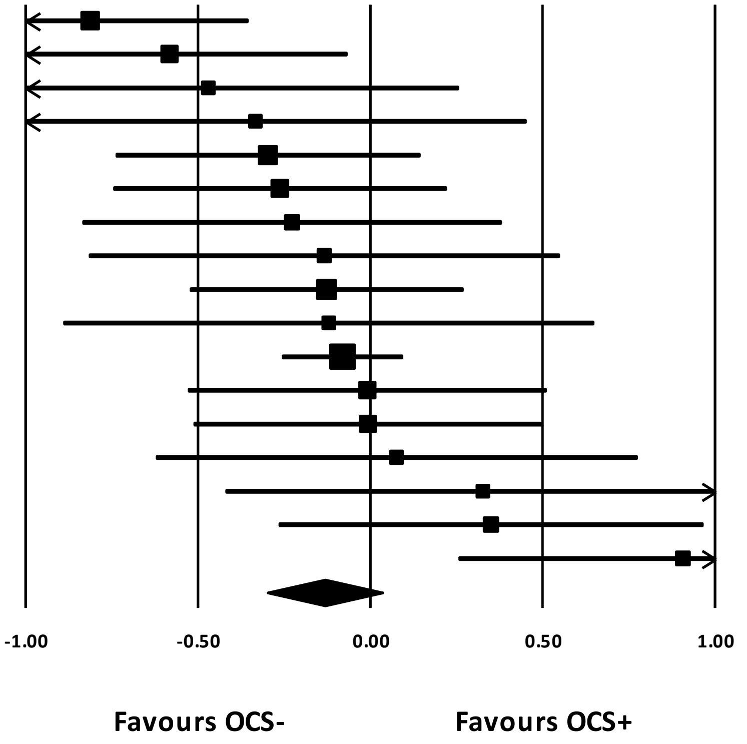

Figure 1 Forest plot processing speed overall

Study name	Subgroup within study	Outcome	Statistics for each study						
			Std diff in means	Standard error	Variance	Lower limit	Upper limit	Z-Value	p-Value
Sahoo 2018	Blank	TMTA percentile	-0.812	0.234	0.055	-1.271	-0.353	-3.469	0.001
Berman 1998	Blank	TMTA	-0.470	0.371	0.138	-1.197	0.257	-1.266	0.205
Manheim study	Blank	TMTA	-0.297	0.225	0.051	-0.738	0.145	-1.315	0.188
Frias 2014	Blank	TMTA	-0.262	0.247	0.061	-0.745	0.222	-1.060	0.289
Kim 2015	Combined	TMTA	-0.126	0.202	0.041	-0.523	0.270	-0.625	0.532
Tiryaki 2010	Blank	TMTA	-0.008	0.265	0.070	-0.529	0.512	-0.032	0.975
Kazhungil 2017	Blank	TMTA	-0.006	0.258	0.067	-0.512	0.500	-0.024	0.981
Borkowska 2013	Blank	TMTA	0.327	0.381	0.146	-0.420	1.075	0.858	0.391
Focseneanu 2016	Blank	TMTA	0.350	0.314	0.099	-0.265	0.966	1.116	0.265
			-0.177	0.117	0.014	-0.406	0.052	-1.518	0.129

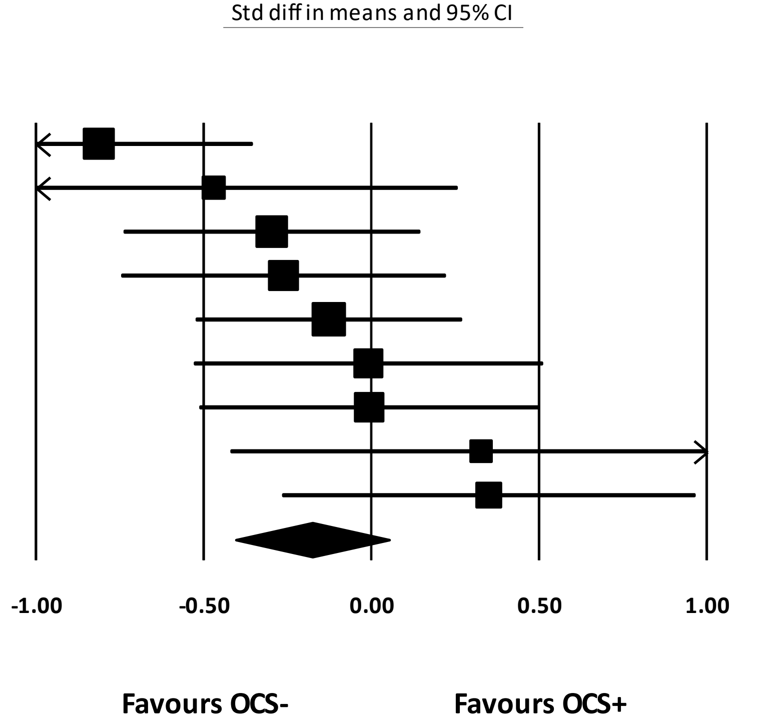

Figure 2 Forest plot processing speed: TMTA only

Study name	Subgroup within study	Outcome	Statistics for each study						
			Std diff in means	Standard error	Variance	Lower limit	Upper limit	Z-Value	p-Value
Focseneanu 2016	Blank	Stroop word	-0.993	0.330	0.109	-1.639	-0.346	-3.010	0.003
Lee 2009	Blank	Stroop simple	-0.333	0.401	0.161	-1.119	0.454	-0.829	0.407
Tiryaki 2010	Blank	Stroop word	-0.256	0.266	0.071	-0.778	0.266	-0.962	0.336
Tumkaya 2009	Blank	Stroop word	-0.227	0.310	0.096	-0.835	0.382	-0.730	0.465
Bleich Cohen	Blank	Nback RT	-0.133	0.349	0.122	-0.817	0.550	-0.382	0.703
Kim 2015	Combined	CPT RT	-0.026	0.202	0.041	-0.422	0.370	-0.129	0.897
Borkowska 2013	Blank	Stroop word	0.037	0.379	0.144	-0.706	0.780	0.097	0.923
Veerman 2016	Blank	RTI 5 choice RT	0.077	0.356	0.127	-0.622	0.776	0.216	0.829
Michalopoulou 2014	Blank	Stroop word	0.907	0.332	0.110	0.256	1.558	2.731	0.006
			-0.105	0.157	0.025	-0.413	0.203	-0.669	0.504

Std diff in means and 95% CI

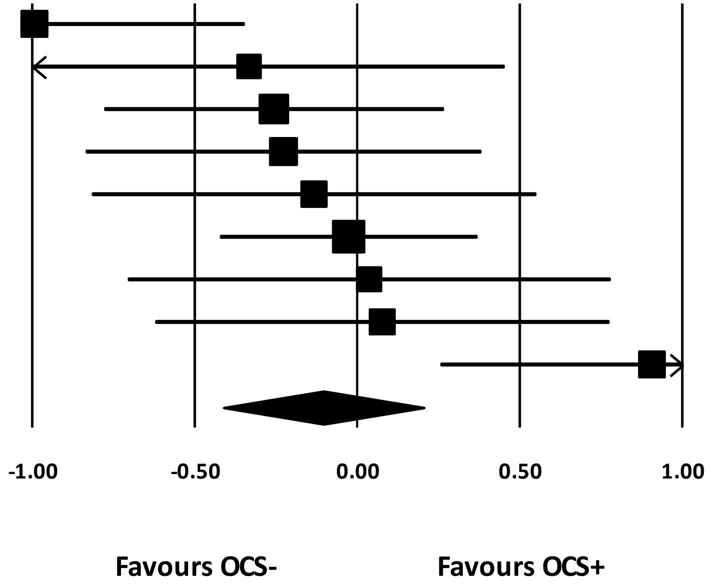

Figure 3 Forest plot processing speed: purely RT based

Study name	Subgroup within study	Outcome	Std diff in means	Standard error	Variance	Lower limit	Upper limit	Z-Value	p-Value
			Statistics for each study						
Sahoo 2018	Blank	TMTA percentile	-0.812	0.234	0.055	-1.271	-0.353	-3.469	0.001
Tonna 2019	Blank	MATRICS	-0.582	0.263	0.069	-1.098	-0.066	-2.212	0.027
Berman 1998	Blank	TMTA	-0.470	0.371	0.138	-1.197	0.257	-1.266	0.205
Manheim study	Blank	TMTA	-0.297	0.225	0.051	-0.738	0.145	-1.315	0.188
Frias 2014	Blank	TMTA	-0.262	0.247	0.061	-0.745	0.222	-1.060	0.289
Kim 2015	Combined	TMTA	-0.126	0.202	0.041	-0.523	0.270	-0.625	0.532
Khumbani 2010	Blank	DKEFS number sequencing	-0.120	0.393	0.154	-0.890	0.650	-0.306	0.760
GROUP study	Blank	WAIS digit symbol substitution raw score	-0.081	0.090	0.008	-0.256	0.095	-0.898	0.369
Tiryaki 2010	Blank	TMTA	-0.008	0.265	0.070	-0.529	0.512	-0.032	0.975
Kazhungil 2017	Blank	TMTA	-0.006	0.258	0.067	-0.512	0.500	-0.024	0.981
Borkowska 2013	Blank	TMTA	0.327	0.381	0.146	-0.420	1.075	0.858	0.391
Focseneanu 2016	Blank	TMTA	0.350	0.314	0.099	-0.265	0.966	1.116	0.265
			-0.190	0.087	0.008	-0.362	-0.019	-2.180	0.029

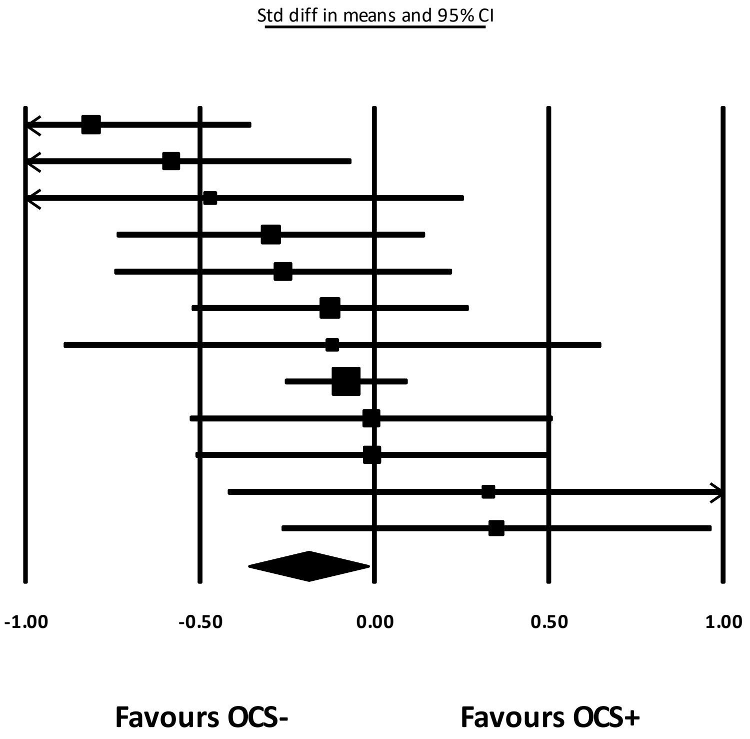

Figure 4 Forest plot processing speed: not purely RT based

Study name	Subgroup within study	Outcome	Statistics for each study						
			Std diff in means	Standard error	Variance	Lower limit	Upper limit	Z-Value	p-Value
Lysaker 2002	Blank	CPT hits	-0.761	0.339	0.115	-1.424	-0.097	-2.245	0.025
Whitney 2003	Blank	CPT attentiveness score	-0.466	0.276	0.076	-1.007	0.075	-1.688	0.091
Lysaker 2004	Combined	CPT omissions	-0.068	0.265	0.070	-0.588	0.452	-0.257	0.797
Manheim study	Blank	CPT # mistakes	-0.068	0.224	0.050	-0.507	0.372	-0.301	0.763
GROUP study	Blank	CPT-HQ accuracy, overall	-0.058	0.092	0.008	-0.238	0.121	-0.635	0.526
Kim 2015	Combined	CPT correct response	0.057	0.202	0.041	-0.339	0.453	0.282	0.778
Veerman 2016	Blank	RVP a	0.185	0.371	0.138	-0.542	0.913	0.499	0.617
			-0.107	0.084	0.007	-0.272	0.058	-1.267	0.205

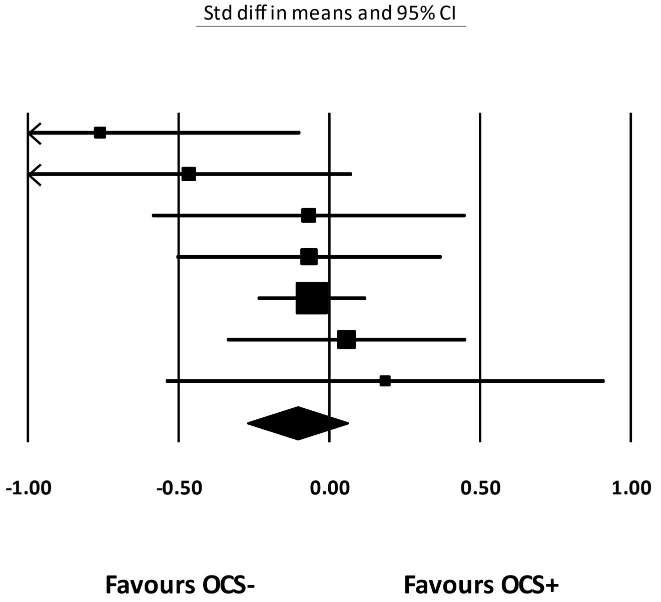

Figure 5 Forest plot sustained attention

c. Forest plots memory

Study name	Outcome	Subgroup within study	Std diff in means	Standard error	Variance	Lower limit	Upper limit	Z-Value	p-Value
			Statistics for each study						
Berman 1998	digit span	Blank	-0.549	0.373	0.139	-1.280	0.182	-1.473	0.141
Hamid 2010	digit span	Blank	-0.543	0.283	0.080	-1.097	0.011	-1.919	0.055
Veerman 2016	SWM	Blank	-0.396	0.359	0.129	-1.099	0.307	-1.105	0.269
Tonna 2019	MATRICS	Blank	-0.318	0.259	0.067	-0.827	0.190	-1.227	0.220
Kim 2015	digit span forward	Combined	-0.314	0.203	0.041	-0.712	0.083	-1.549	0.121
Manheim study	nback# mistakes	Blank	-0.102	0.224	0.050	-0.541	0.338	-0.453	0.651
Frias 2014	digit span	Blank	-0.083	0.246	0.060	-0.565	0.399	-0.338	0.735
GROUP	arithemetic	Blank	-0.056	0.090	0.008	-0.233	0.120	-0.626	0.531
Kazhungil 2017	WMSLNS	Blank	0.013	0.258	0.067	-0.493	0.519	0.051	0.959
Kontis 2016	SWM	Blank	0.095	0.213	0.046	-0.324	0.513	0.444	0.657
Michalopoulou 2014	digit span	Blank	0.105	0.316	0.100	-0.515	0.725	0.333	0.739
Tumkaya 2009	digit span	Blank	0.167	0.310	0.096	-0.441	0.775	0.538	0.590
Bleich cohen 2014	Nback accuracy	Blank	0.418	0.352	0.124	-0.272	1.108	1.187	0.235
Wang 2019	Digit span forward	Blank	0.618	0.316	0.100	-0.002	1.237	1.953	0.051
Lee 2009	arithemetic	Blank	1.235	0.433	0.187	0.387	2.083	2.856	0.004
			-0.030	0.087	0.008	-0.201	0.141	-0.346	0.729

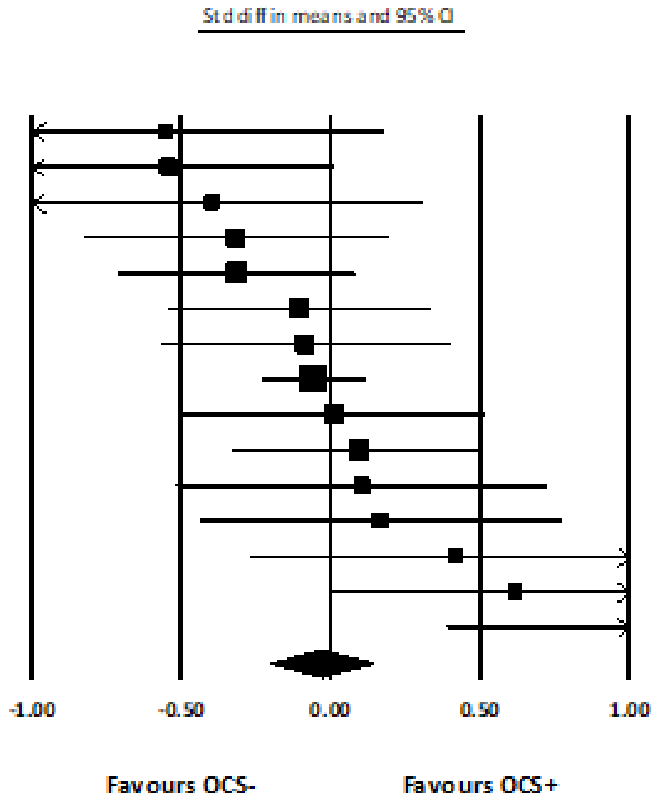

Figure 6 Forest plot working memory

Study name	Outcome	Subgroup within study	Std diff in means	Standard error	Variance	Lower limit	Upper limit	Z-Value	p-Value
			Statistics for each study						
Berman 1998	digit span	Blank	-0.549	0.373	0.139	-1.280	0.182	-1.473	0.141
Hamid 2010	digit span	Blank	-0.543	0.283	0.080	-1.097	0.011	-1.919	0.055
Kim 2015	digit span forward	Combined	-0.314	0.203	0.041	-0.712	0.083	-1.549	0.121
Frias 2014	digit span	Blank	-0.083	0.246	0.060	-0.565	0.399	-0.338	0.735
Michalopoulou 2014	digit span	Blank	0.105	0.316	0.100	-0.515	0.725	0.333	0.739
Tumkaya 2009	digit span	Blank	0.167	0.310	0.096	-0.441	0.775	0.538	0.590
Wang 2019	Digit span forward	Blank	0.618	0.316	0.100	-0.002	1.237	1.953	0.051
			-0.097	0.148	0.022	-0.386	0.192	-0.658	0.511

Std diff in means and 95% CI

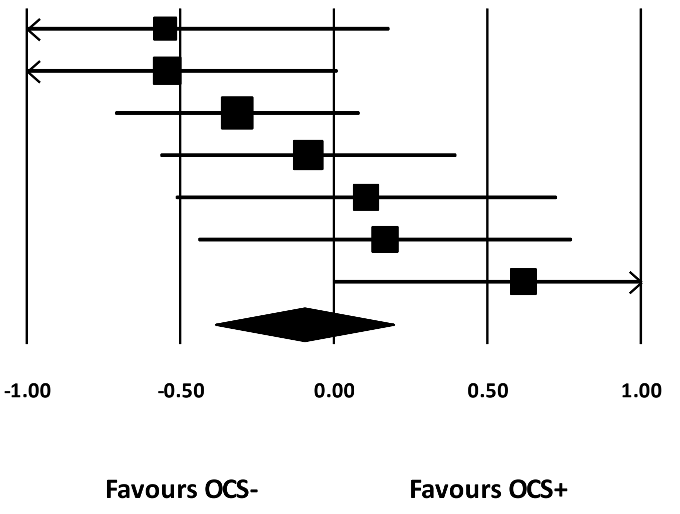

Figure 7 Forest plot working memory: digit span

Study name	Subgroup within study	Outcome	Std diff in means	Standard error	Variance	Lower limit	Upper limit	Z-Value	p-Value
			Statistics for each study						
Manheim study	Blank	RCFT memory	-0.706	0.231	0.053	-1.159	-0.253	-3.057	0.002
Veerman 2016	Blank	PAL total errors adjusted	-0.280	0.357	0.127	-0.979	0.418	-0.787	0.432
Whitney 2003	Blank	RCFT percent recalled	-0.234	0.273	0.075	-0.770	0.302	-0.856	0.392
Tonna 2019	Blank	MATRICS	-0.191	0.258	0.067	-0.697	0.316	-0.739	0.460
Kazhungil 2017	Blank	RCFT immediate	-0.107	0.258	0.067	-0.613	0.400	-0.413	0.680
Michalopoulou 2014	Blank	RCFT immediate	-0.084	0.316	0.100	-0.704	0.536	-0.265	0.791
Berman 1998	Blank	immediate visual memory	0.024	0.366	0.134	-0.693	0.741	0.065	0.948
Tumkaya 2009	Blank	WMS immediate	0.064	0.310	0.096	-0.543	0.670	0.205	0.837
Lee 2009	Blank	RCFT immediate	0.282	0.400	0.160	-0.503	1.067	0.704	0.481
Kontis 2016	Blank	PRM	0.319	0.209	0.044	-0.090	0.729	1.527	0.127
Lysaker 2002	Blank	VRT	0.860	0.341	0.116	0.193	1.528	2.526	0.012
			-0.030	0.126	0.016	-0.277	0.216	-0.241	0.810

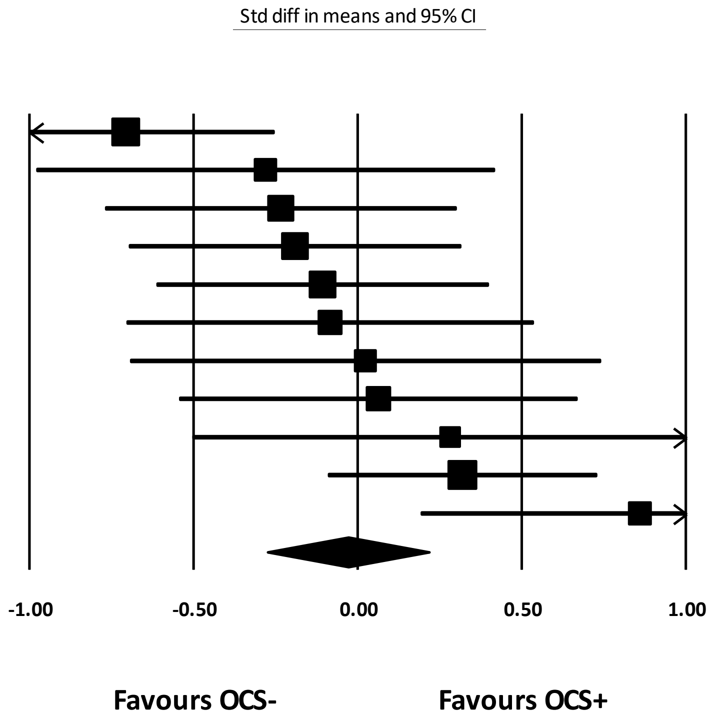

Figure 8 Forest plot immediate visual memory

Study name	Subgroup within study	Outcome	Std diff in means	Standard error	Variance	Lower limit	Upper limit	Z-Value	p-Value
Berman 1998	Blank	delayed visual memory	-0.440	0.370	0.137	-1.165	0.286	-1.187	0.235
Tumkaya 2009	Blank	WMS delayed	0.113	0.310	0.096	-0.495	0.720	0.364	0.716
Kazhungil 2017	Blank	RCFT delayed	0.181	0.259	0.067	-0.326	0.688	0.700	0.484
Lee 2009	Blank	RCFT delayed	0.210	0.400	0.160	-0.573	0.993	0.526	0.599
			0.051	0.160	0.026	-0.263	0.365	0.319	0.749

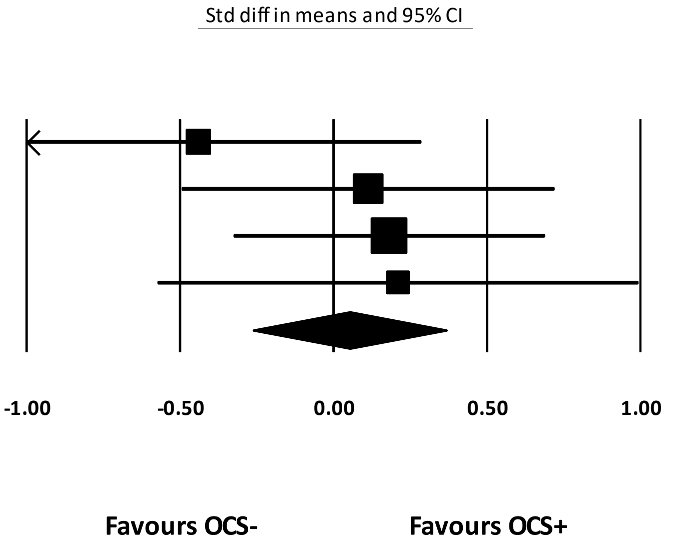

Figure 9 Forest plot delayed visual memory

Study name	Subgroup within study	Outcome	Std diff in means	Standard error	Variance	Lower limit	Upper limit	Z-Value	p-Value
			Statistics for each study						
Hamid 2010	Blank	RAVLT trial 1	-0.425	0.282	0.079	-0.977	0.127	-1.510	0.131
Kim 2015	Combined	RAVLT A1	-0.234	0.203	0.041	-0.631	0.163	-1.157	0.247
Kazhungil 2017	Blank	RAVLT immediate recall	0.169	0.259	0.067	-0.338	0.676	0.653	0.514
Veerman 2016	Blank	VRM phase 1	0.490	0.358	0.128	-0.211	1.192	1.370	0.171
Tumkaya 2009	Blank	RAVLT short term memory	0.689	0.318	0.101	0.066	1.312	2.168	0.030
Lee 2009	Blank	RAVLT trial 1	1.040	0.423	0.179	0.211	1.869	2.460	0.014
			0.224	0.214	0.046	-0.195	0.643	1.048	0.295

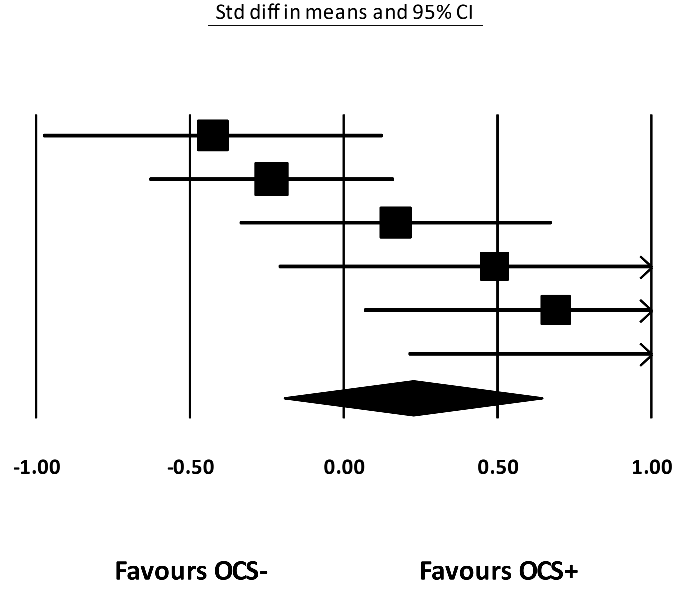

Figure 10 Forest plot trial 1 verbal memory

Study name	Subgroup within study	Outcome	Statistics for each study							Std diff in means and 95% CI
			**Std diff in means**	**Standard error**	**Variance**	**Lower limit**	**Upper limit**	**Z-Value**	**p-Value**	
Manheim study	Blank	RAVLT immediate recall	-0.381	0.226	0.051	-0.824	0.063	-1.683	0.092	
Whitney 2003	Blank	CVLT	-0.220	0.273	0.075	-0.755	0.316	-0.804	0.422	
Tonna 2019	Blank	MATRICS	-0.148	0.258	0.067	-0.654	0.358	-0.574	0.566	
GROUP study	Blank	15WLT total items correct immediate recall	0.049	0.090	0.008	-0.126	0.225	0.551	0.581	
Focseneanu 2016	Blank	RAVLT sum of tests	0.636	0.319	0.102	0.010	1.262	1.993	0.046	
			-0.035	0.136	0.019	-0.302	0.232	-0.256	0.798	

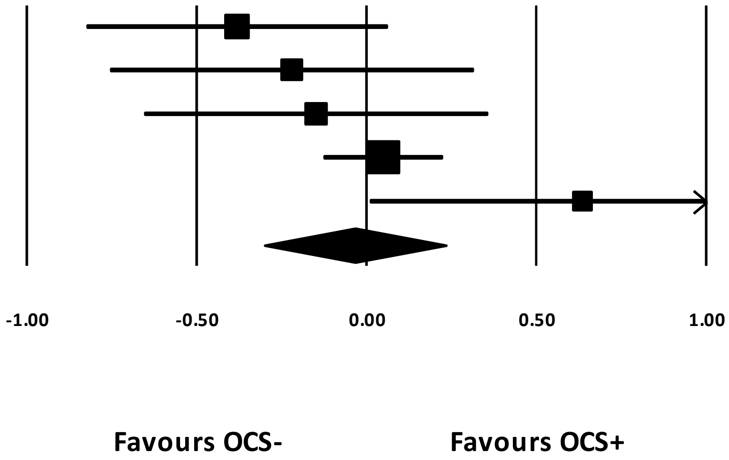

Figure 11 Forest plot sum of tests verbal memory

Study name	Subgroup within study	Outcome	Statistics for each study						
			Std diff in means	Standard error	Variance	Lower limit	Upper limit	Z-Value	p-Value
Tumkaya 2009	Blank	RAVLT delayed free recall	-0.203	0.310	0.096	-0.811	0.405	-0.653	0.514
Manheim study	Blank	RAVLT delayed recall	-0.200	0.225	0.051	-0.641	0.241	-0.890	0.374
Lee 2009	Blank	RAVLT delayed recall	-0.198	0.399	0.160	-0.981	0.585	-0.495	0.620
GROUP	Blank	WLT delayed items correct	0.035	0.090	0.008	-0.141	0.211	0.387	0.699
Kim 2015	Combined	RAVLT delayed	0.167	0.202	0.041	-0.229	0.564	0.828	0.408
Kazhungil 2017	Blank	RAVLT delayed recall	0.241	0.259	0.067	-0.267	0.749	0.931	0.352
			0.023	0.071	0.005	-0.115	0.162	0.332	0.740

Std diff in means and 95% CI

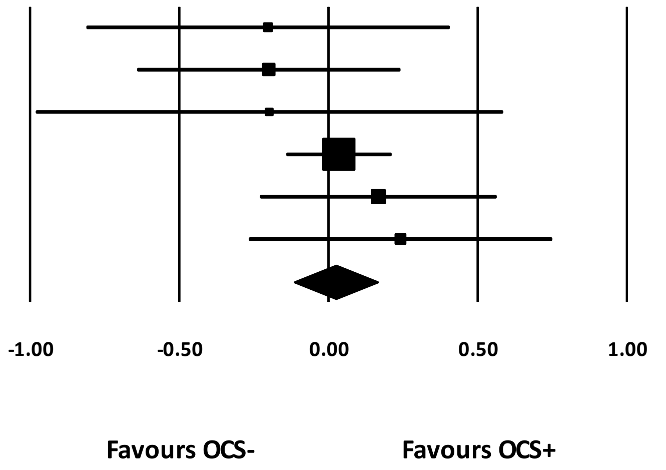

Figure 12 Forest plot delayed verbal memory

d. Forest plots executive function

Study name	Subgroup within study	Outcome	Std diff in means	Standard error	Variance	Lower limit	Upper limit	Z-Value	p-Value
Sahoo 2018	Blank	COWA new words percentile	-1.059	0.240	0.058	-1.530	-0.588	-4.406	0.000
Whitton 2013	Blank	fluency	-0.797	0.387	0.149	-1.554	-0.039	-2.060	0.039
Michalopoulou 2014	Blank	COWA	-0.200	0.317	0.100	-0.821	0.421	-0.631	0.528
Berman 1998	Blank	FAS	-0.056	0.366	0.134	-0.774	0.661	-0.154	0.877
Kazhungil 2017	Blank	COWA	-0.027	0.258	0.067	-0.533	0.479	-0.104	0.917
Tiryaki 2010	Blank	Combined	-0.025	0.266	0.071	-0.546	0.496	-0.092	0.926
Borkowska 2003	Blank	VF	0.000	0.379	0.144	-0.743	0.743	0.000	1.000
Frias 2014	Blank	FAS	0.043	0.246	0.060	-0.439	0.524	0.174	0.862
Lee 2009	Blank	word fluency	1.336	0.438	0.192	0.477	2.194	3.049	0.002
			-0.123	0.198	0.039	-0.512	0.265	-0.622	0.534

Statistics for each study

Std diff in means and 95% CI

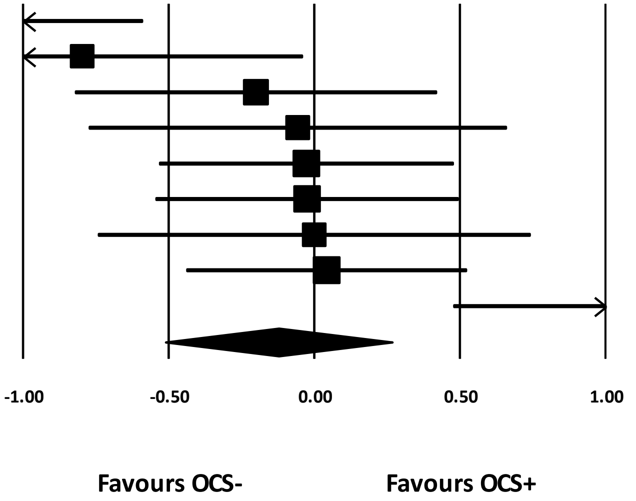

Figure 13 Forest plot fluency

Study name	Subgroup within study	Outcome	Statistics for each study						
			Std diff in means	Standard error	Variance	Lower limit	Upper limit	Z-Value	p-Value
Ongur 2005	Blank	Stroop interference	-1.116	0.294	0.086	-1.692	-0.540	-3.799	0.000
Lee 2009	Blank	Stroop interference	-1.002	0.421	0.177	-1.828	-0.177	-2.379	0.017
Manheim study	Blank	Stroop interference	-0.435	0.227	0.051	-0.879	0.010	-1.916	0.055
Whitton 2013	Blank	HSCT	-0.221	0.361	0.131	-0.930	0.487	-0.612	0.540
Tiryaki 2010	Blank	Stroop 3	-0.143	0.266	0.071	-0.664	0.378	-0.538	0.590
Sahoo 2018	Blank	Stroop effect percentile	0.039	0.225	0.051	-0.402	0.480	0.175	0.861
Tumkaya 2009	Blank	Stroop interference	0.089	0.310	0.096	-0.518	0.696	0.287	0.774
Borkowska 2003	Blank	Stroop interference	0.105	0.385	0.149	-0.650	0.861	0.273	0.785
Focseneanu 2016	Blank	Stroop interference	0.189	0.313	0.098	-0.424	0.801	0.604	0.546
Kazhungil 2017	Blank	Stroop interference	0.222	0.259	0.067	-0.285	0.730	0.859	0.391
			-0.208	0.144	0.021	-0.489	0.074	-1.446	0.148

Std diff in means and 95% CI

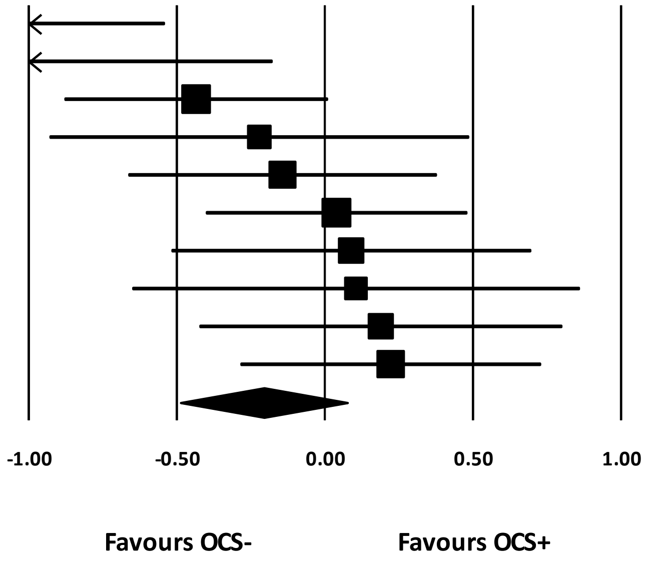

Figure 14 Forest plot cognitive inhibition

Study name	Subgroup within study	Outcome	Statistics for each study						
			Std diff in means	Standard error	Variance	Lower limit	Upper limit	Z-Value	p-Value
Ongur 2005	Blank	Stroop interference	-1.116	0.294	0.086	-1.692	-0.540	-3.799	0.000
Lee 2009	Blank	Stroop interference	-1.002	0.421	0.177	-1.828	-0.177	-2.379	0.017
Manheim study	Blank	Stroop interference	-0.435	0.227	0.051	-0.879	0.010	-1.916	0.055
Tiryaki 2010	Blank	Stroop 3	-0.143	0.266	0.071	-0.664	0.378	-0.538	0.590
Sahoo 2018	Blank	Stroop effect percentile	0.039	0.225	0.051	-0.402	0.480	0.175	0.861
Tumkaya 2009	Blank	Stroop interference	0.089	0.310	0.096	-0.518	0.696	0.287	0.774
Borkowska 2003	Blank	Stroop interference	0.105	0.385	0.149	-0.650	0.861	0.273	0.785
Focseneanu 2016	Blank	Stroop interference	0.189	0.313	0.098	-0.424	0.801	0.604	0.546
Kazhungil 2017	Blank	Stroop interference	0.222	0.259	0.067	-0.285	0.730	0.859	0.391
			-0.208	0.157	0.025	-0.516	0.100	-1.321	0.186

Std diff in means and 95% CI

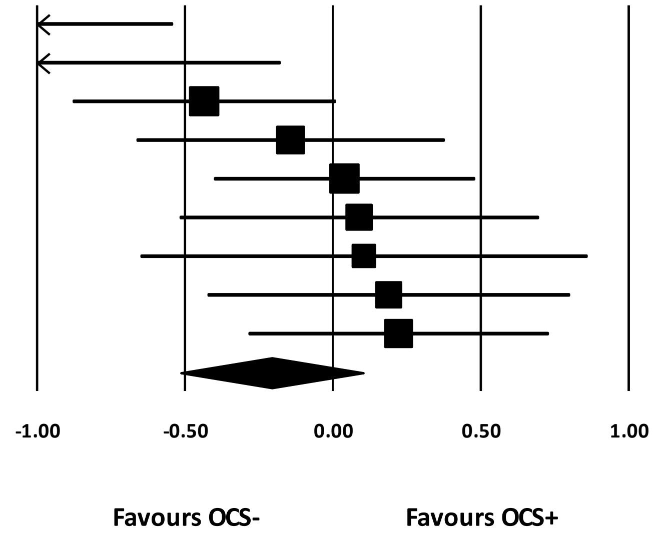

Figure 15 Forest plot cognitive inhibition: Stroop only

Study name	Subgroup within study	Outcome	Std diff in means	Standard error	Variance	Lower limit	Upper limit	Z-Value	p-Value
			**Statistics for each study**						
Ongur 2005	Blank	TMTB seconds	-1.643	0.304	0.092	-2.239	-1.047	-5.403	0.000
Sahoo 2018	Blank	TMTB percentile	-0.852	0.235	0.055	-1.313	-0.392	-3.626	0.000
Manheim study	Blank	TMTB seconds	-0.467	0.227	0.052	-0.912	-0.022	-2.055	0.040
Berman 1998	Blank	TMTB seconds	-0.460	0.371	0.137	-1.186	0.267	-1.240	0.215
Frias 2014	Blank	TMTB seconds	-0.230	0.246	0.061	-0.713	0.253	-0.934	0.350
Kazhungil 2017	Blank	TMTB seconds	-0.123	0.258	0.067	-0.630	0.384	-0.476	0.634
Kim 2015	Combined	TMTB seconds	-0.063	0.203	0.041	-0.461	0.334	-0.313	0.754
Tiryaki 2010	Blank	TMTB seconds	0.237	0.266	0.071	-0.285	0.759	0.889	0.374
Kumbhani 2010	Blank	DKEFS trial switching	0.262	0.396	0.156	-0.513	1.038	0.663	0.507
Tumkaya 2009	Blank	TMT B-A	0.295	0.311	0.097	-0.314	0.905	0.950	0.342
Borkowska 2003	Blank	TMTB seconds	0.460	0.384	0.147	-0.292	1.213	1.199	0.231
Focseneanu 2016	Blank	TMTB seconds	1.013	0.330	0.109	0.365	1.660	3.065	0.002
			-0.150	0.183	0.033	-0.508	0.208	-0.820	0.412

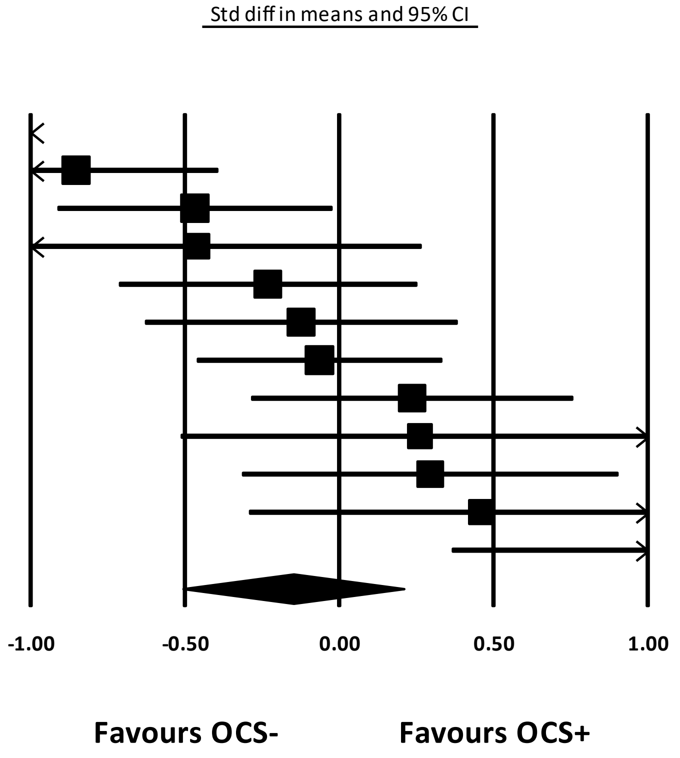

Figure 16 Forest plot cognitive flexibility

Study name	Subgroup within study	Outcome	Std diff in means	Standard error	Variance	Lower limit	Upper limit	Z-Value	p-Value
			Statistics for each study						
Ongur 2005	Blank	TMTB seconds	-1.643	0.304	0.092	-2.239	-1.047	-5.403	0.000
Manheim study	Blank	TMTB seconds	-0.467	0.227	0.052	-0.912	-0.022	-2.055	0.040
Berman 1998	Blank	TMTB seconds	-0.460	0.371	0.137	-1.186	0.267	-1.240	0.215
Frias 2014	Blank	TMTB seconds	-0.230	0.246	0.061	-0.713	0.253	-0.934	0.350
Kazhungil 2017	Blank	TMTB seconds	-0.123	0.258	0.067	-0.630	0.384	-0.476	0.634
Kim 2015	Combined	TMTB seconds	-0.063	0.203	0.041	-0.461	0.334	-0.313	0.754
Tiryaki 2010	Blank	TMTB seconds	0.237	0.266	0.071	-0.285	0.759	0.889	0.374
Borkowska 2003	Blank	TMTB seconds	0.460	0.384	0.147	-0.292	1.213	1.199	0.231
Focseneanu 2016	Blank	TMTB seconds	1.013	0.330	0.109	0.365	1.660	3.065	0.002
			-0.152	0.216	0.047	-0.575	0.271	-0.704	0.482

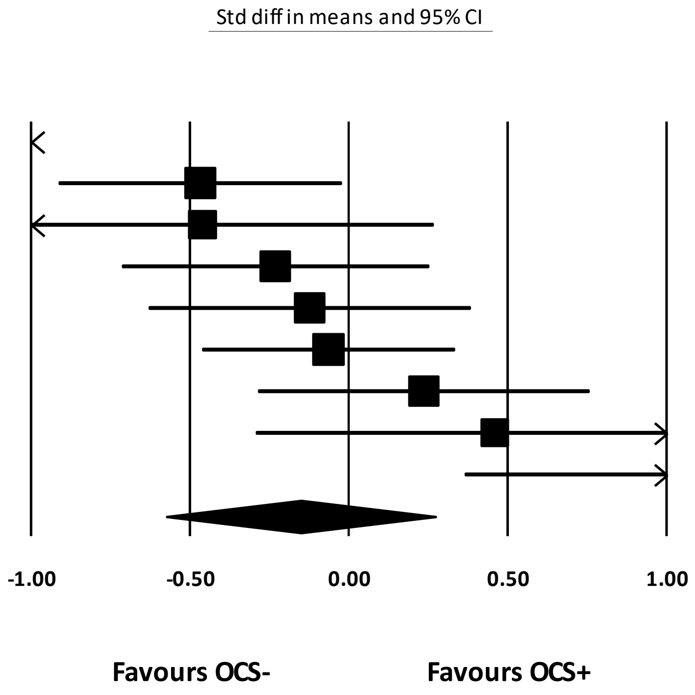

Figure 17 Forest plot cognitive flexibility: TMTB seconds

Study name	Subgroup within study	Outcome	Std diff in means	Standard error	Variance	Lower limit	Upper limit	Z-Value	p-Value
			Statistics for each study						
Hwang 2000	Blank	WCST perseverative errors	-1.262	0.490	0.240	-2.222	-0.302	-2.577	0.010
Patel 2010	Blank	IED	-1.001	0.405	0.164	-1.794	-0.208	-2.474	0.013
Manheim study	Blank	WCST perseverative errors	-0.811	0.233	0.054	-1.268	-0.354	-3.477	0.001
Michalopoulou 2014	Blank	WCST perseverative errors	-0.540	0.322	0.104	-1.170	0.091	-1.676	0.094
Hermesh 2003	Blank	WCST perseverative errors	-0.445	0.321	0.103	-1.073	0.183	-1.388	0.165
Kumbhani 2010	Blank	WCST perseverative errors	-0.345	0.398	0.158	-1.125	0.435	-0.867	0.386
Lysaker 2000	Blank	WCST perseverative errors	-0.139	0.296	0.088	-0.720	0.441	-0.471	0.638
GROUP study	Blank	RST cost score index	0.007	0.093	0.009	-0.176	0.189	0.071	0.944
Kontis 2016	Blank	IED	0.035	0.211	0.044	-0.378	0.448	0.168	0.866
Whitney 2003	Blank	WCST perseverative errors	0.144	0.273	0.074	-0.390	0.679	0.529	0.597
Kazhungil 2017	Blank	WCST perseverative errors	0.297	0.260	0.067	-0.212	0.806	1.143	0.253
Tumkaya 2009	Blank	WCST perseverative errors	0.499	0.314	0.099	-0.116	1.115	1.591	0.112
Ongur 2005	Blank	WCST perseverative errors	1.535	0.302	0.091	0.944	2.126	5.087	0.000
			-0.111	0.162	0.026	-0.429	0.206	-0.688	0.492

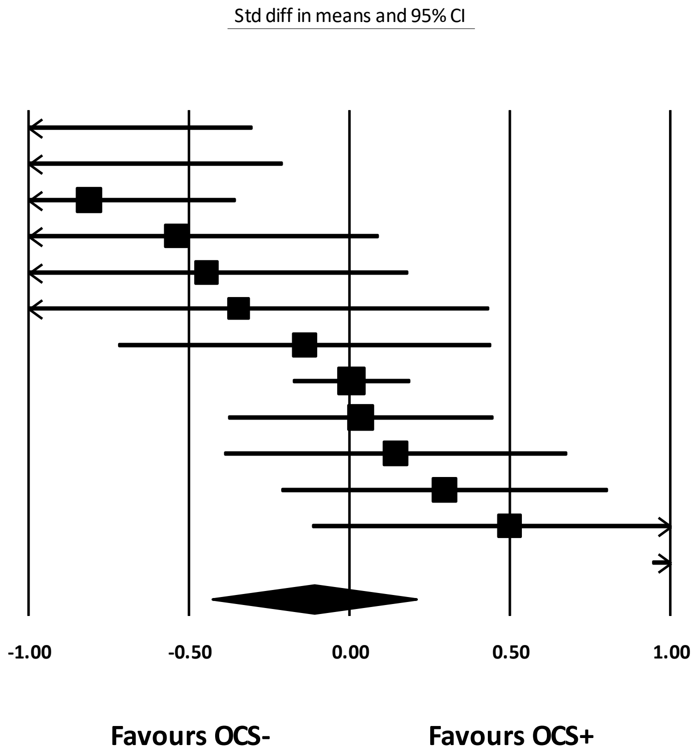

Figure 18 Forest plot set shifting

Study name	Subgroup within study	Outcome	Statistics for each study						
			Std diff in means	Standard error	Variance	Lower limit	Upper limit	Z-Value	p-Value
Hwang 2000	Blank	WCST perseverative errors	-1.262	0.490	0.240	-2.222	-0.302	-2.577	0.010
Manheim study	Blank	WCST perseverative errors	-0.811	0.233	0.054	-1.268	-0.354	-3.477	0.001
Michalopoulou 2014	Blank	WCST perseverative errors	-0.540	0.322	0.104	-1.170	0.091	-1.676	0.094
Hermesh 2003	Blank	WCST perseverative errors	-0.445	0.321	0.103	-1.073	0.183	-1.388	0.165
Kumbhani 2010	Blank	WCST perseverative errors	-0.345	0.398	0.158	-1.125	0.435	-0.867	0.386
Lysaker 2000	Blank	WCST perseverative errors	-0.139	0.296	0.088	-0.720	0.441	-0.471	0.638
Whitney 2003	Blank	WCST perseverative errors	0.144	0.273	0.074	-0.390	0.679	0.529	0.597
Kazhungil 2017	Blank	WCST perseverative errors	0.297	0.260	0.067	-0.212	0.806	1.143	0.253
Tumkaya 2009	Blank	WCST perseverative errors	0.499	0.314	0.099	-0.116	1.115	1.591	0.112
Ongur 2005	Blank	WCST perseverative errors	1.535	0.302	0.091	0.944	2.126	5.087	0.000
			-0.081	0.239	0.057	-0.549	0.387	-0.338	0.735

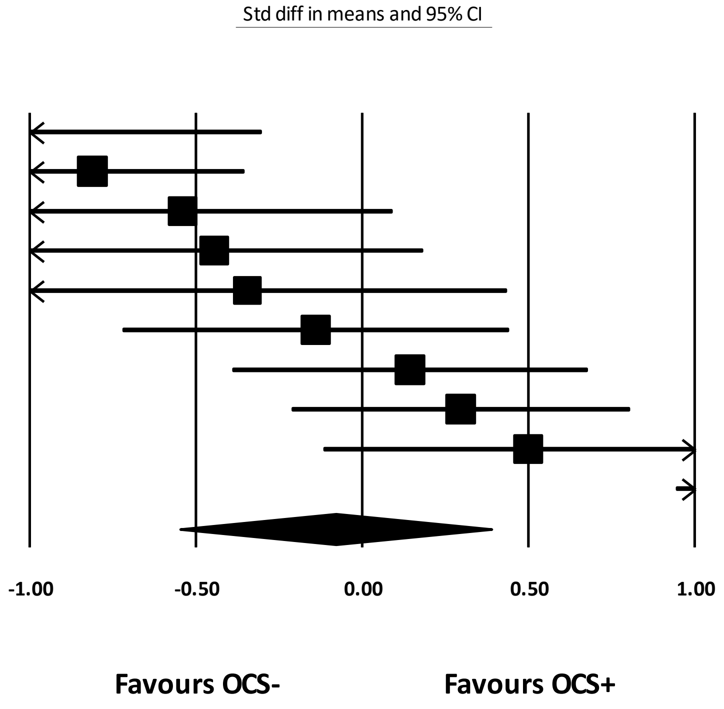

Figure 19 Forest plot set shifting: WCST

Study name	Subgroup within study	Outcome	Std diff in means	Standard error	Variance	Lower limit	Upper limit	Z-Value	p-Value
			Statistics for each study						
Hwang 2000	Blank	WCST categories completed	-1.105	0.480	0.231	-2.046	-0.164	-2.302	0.021
Lysaker 2004	Combined	WCST categories completed	-0.872	0.275	0.076	-1.411	-0.333	-3.172	0.002
Manheim study	Blank	WCST categories completed	-0.413	0.227	0.051	-0.857	0.031	-1.823	0.068
Lysaker 2000	Blank	WCST categories completed	-0.366	0.298	0.089	-0.951	0.219	-1.227	0.220
Kazhungil 2017	Blank	WCST categories completed	-0.197	0.259	0.067	-0.705	0.310	-0.763	0.446
Michalopoulou 2014	Blank	WCST categories completed	-0.150	0.317	0.100	-0.770	0.471	-0.473	0.636
Ongur 2005	Blank	WCST categories completed	-0.127	0.285	0.081	-0.686	0.431	-0.448	0.654
Berman 1998	Blank	WCST categories completed	-0.038	0.366	0.134	-0.756	0.679	-0.105	0.916
Hermesh 2003	Blank	WCST categories completed	0.095	0.317	0.100	-0.526	0.716	0.301	0.763
Kumbhani 2010	Blank	WCST categories completed	0.100	0.393	0.154	-0.670	0.870	0.255	0.799
Kim 2015	Combined	WCST categories completed	0.207	0.202	0.041	-0.190	0.604	1.023	0.306
Tumkaya 2009	Blank	WCST categories completed	0.578	0.315	0.099	-0.040	1.197	1.834	0.067
			-0.168	0.122	0.015	-0.407	0.071	-1.375	0.169

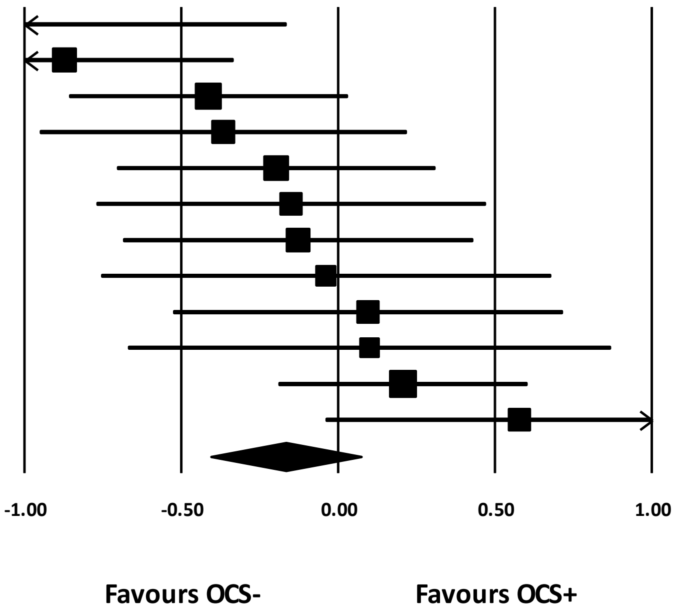

Figure 20 Forest plot abstract thinking

Study name	Subgroup within study	Outcome	Statistics for each study						
			Std diff in means	Standard error	Variance	Lower limit	Upper limit	Z-Value	p-Value
Sahoo 2018	Blank	ToL 3 minmoves	-0.605	0.230	0.053	-1.056	-0.154	-2.627	0.009
Patel 2010	Blank	SoC	-0.580	0.390	0.152	-1.344	0.184	-1.489	0.137
Veerman 2016	Blank	OTS	-0.280	0.357	0.127	-0.978	0.419	-0.784	0.433
Kontis 2016	Blank	SoC	0.447	0.223	0.050	0.010	0.884	2.003	0.045
			-0.229	0.293	0.086	-0.802	0.345	-0.782	0.434

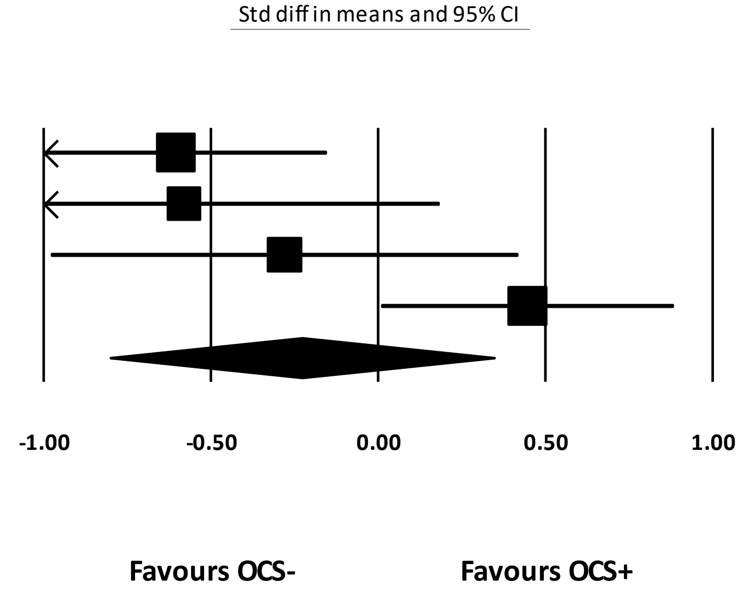

Figure 21 Forest plot planning

Study name	Subgroup within study	Outcome	Std diff in means	Standard error	Variance	Lower limit	Upper limit	Z-Value	p-Value
			Statistics for each study						
Wang 2019	Blank	WAIS similarities	-1.143	0.333	0.111	-1.796	-0.490	-3.431	0.001
Berman 1998	Blank	WAIS similarities	-0.937	0.385	0.149	-1.693	-0.182	-2.431	0.015
Hermesh 2003	Blank	Raven	-0.382	0.319	0.102	-1.009	0.244	-1.197	0.231
Tonna 2019	Blank	MATRICS mazes	-0.000	0.258	0.066	-0.505	0.505	-0.001	0.999
Lee 2009	Blank	WAIS picture arrangement	0.308	0.401	0.161	-0.478	1.093	0.768	0.443
Kazhungil 2017	Blank	WAIS color matrics	0.362	0.260	0.068	-0.148	0.872	1.391	0.164
			-0.281	0.253	0.064	-0.776	0.214	-1.114	0.265

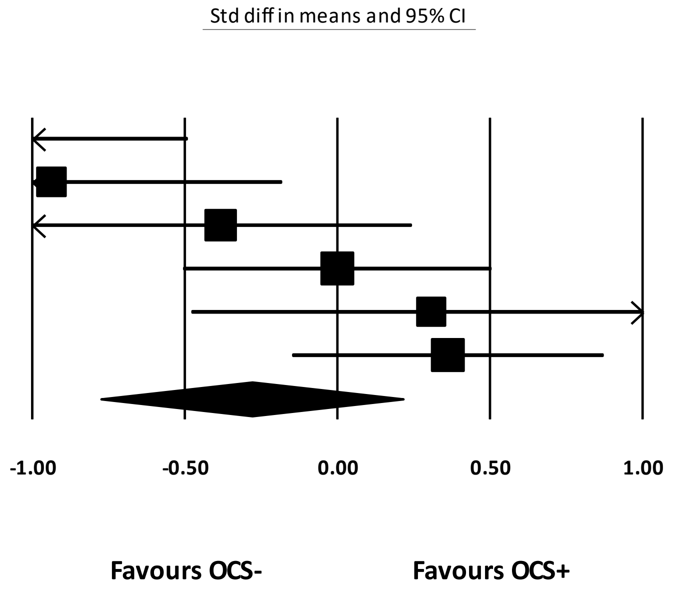

Figure 22 Forest plot reasoning

e. Forest plot facial affect recognition

Study name	Subgroup within study	Outcome	Statistics for each study: Std diff in means	Standard error	Variance	Lower limit	Upper limit	Z-Value	p-Value
Whitton 2013	Blank	Ekman 60 faces	-1.008	0.402	0.162	-1.796	-0.219	-2.505	0.012
GROUP study	Blank	DFAR	-0.030	0.092	0.008	-0.211	0.150	-0.330	0.741
Thessaloniki study	Blank	facial affect perception	0.052	0.252	0.063	-0.441	0.545	0.207	0.836
Manheim study	Blank	emotion recognition	0.055	0.327	0.107	-0.586	0.695	0.167	0.867
Veerman 2016	Blank	emotion recognition	0.056	0.449	0.201	-0.823	0.935	0.125	0.900
			-0.093	0.140	0.020	-0.367	0.182	-0.663	0.507

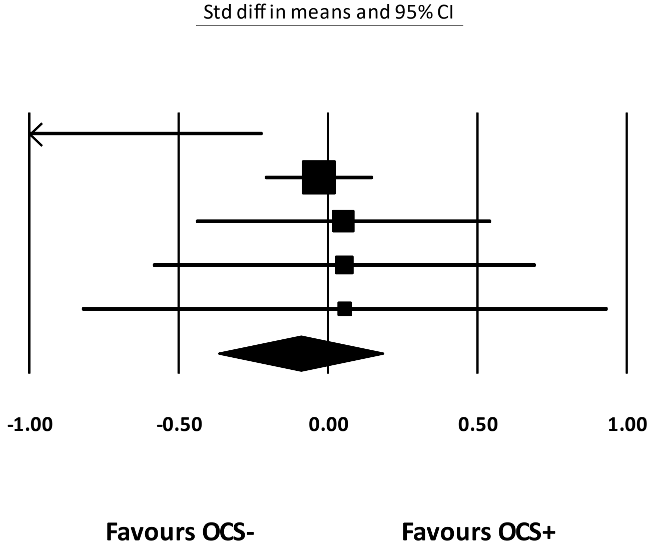

Figure 23 Forest plot facial affect recognition

f. Forest plot visual spatial ability

Study name	Subgroup within study	Outcome	Statistics for each study						
			Std diff in means	Standard error	Variance	Lower limit	Upper limit	Z-Value	p-Value
Manheim study	Blank	block design	-0.483	0.227	0.052	-0.929	-0.038	-2.125	0.034
Frias 2014	Blank	block design	-0.205	0.246	0.061	-0.688	0.278	-0.832	0.406
Berman 1998	Blank	block design	-0.113	0.366	0.134	-0.831	0.605	-0.308	0.758
GROUP study	Blank	block design	-0.042	0.090	0.008	-0.219	0.134	-0.471	0.638
Michalopoulou 2014	Blank	block design	-0.035	0.291	0.084	-0.605	0.535	-0.121	0.904
Lee 2009	Blank	block design	1.263	0.434	0.188	0.413	2.114	2.910	0.004
			-0.038	0.160	0.026	-0.352	0.275	-0.241	0.810

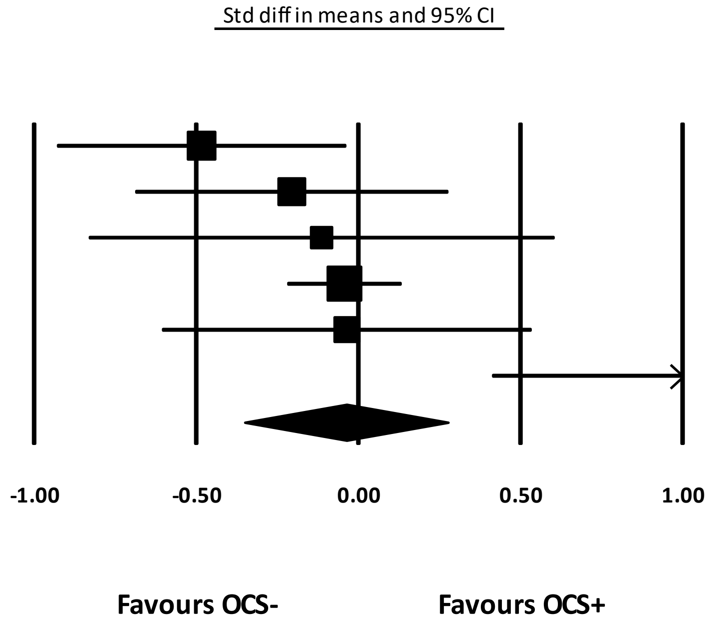

Figure 24 Forest plot visual spatial ability

7. Confounders per subgroup

Below you will find a descriptive table of the confounders age, percentage males, mean PANSS positive score, percentage on clozapine and mean YBOCS for the OCS- and OCS+ group within each study separately. Note that not all of these values can be found in the original publication, as some were provided to us by the authors.

Author & Year	Subgroup	Age	SD	% male	PANSS positive	SD	% on clozapine	YBOCS score	SD
Berman 1998	OCS+	52.1	10.7		22.1	5.6		19.9	6.3
	OCS-	49.4	11.7		19.8	6.3		0	
Bleich-Cohen 2014	OCS+	27.3	3.4	62.5			0	20.2	8.2
	OCS-	25.7	2.3	64.7			0	0.2	0.7
Borkowska 2003	OCS+	26	6	61.5				23	4
	OCS-	33	10	53.3					
Focseneanu 2016	OCS+	23.64	1.76	47.1	19.88	5.94		23.11	6.06
	OCS-	23.3	2.39	19.2	26.57	4.47		0	
Frias 2014	OCS+	37.03	14.40	53.3	16.63	7.01	16.7	18.67	7.54
	OCS-	32.41	10.09	59.5	15.49	5.03	5.4		
GROUP study	OCS+	26.46	7.98	76.8	16.79	6.96	21.9	14.64	5.54
	OCS-	27.78	7.97	76.0	13.44	6.37	12.2	0.34	1.34
Hamid 2010	OCS+	31.47	12.2	73.3					
	OCS-	36.73	9.7	58.8					
Hermesh 2003	OCS+	31.7	10.4	100				21.5	7.3
	OCS-	32.5	13.4	100					
Hwang 2000	OCS+	35.7		90	24.1	4.8		27.9	3.4
	OCS-	38.3		90	23.3	7.1		1.0	1.3
Kazhungil 2017	OCS+	29.1	7.2	56.7	17.93	6.30	13	24.40	4.91
	OCS-	28.7	7.1	56.7	23.20	5.47	0		
Kumbhani 2010	All	40.69	10.79	51.7					
Kim 2015	OCS+ <5	24.6	5.4	38.5	19.5	6.3			
	OCS+>5	37.8	7.0	41.2	21.6	7.1			
	OCS- <5	29.6	8.2	56.6	15	6.4			
	OCS- >5	36.7	8.0	51.2	17.3	6.2			
Kontis 2016	OCS+	40.48	9.19	63.6	18.00	6.84	18.2	18.09	6.45
	OCS-	43.57	10.45	63.6	18.70	9.27	9.1	1.14	2.38
Lee 2009	OCS+	30.30	7.79	70	13.40	3.71	40	14.00	6.34
	OCS-	30.47	5.39	58.8	12.41	3.97	35.3		
Lysaker 2000	OCS+	39.1	10.2	71.4	20.4	6.3			
	OCS-	40.8	9.7	92.0	15.3	5.7			
Lysaker 2002	OCS+							22	4
	OCS-							2.9	5
Lysaker 2004	OCS+ good function	46	11	100	21	4.2		18	6.6

	OCS+ poor function	49	8	100	18	5.7		21	5.4
	OCS- moderate function	44	9	100	17.7	6.9		2	3.1
	OCS- poor function	45	9	100	19	4.2		2	3.2
Manheim study	OCS+	39.7	10.8	73.0	13.9	2.7	59.5	16.5	5.7
	OCS-	35.8	10.0	67.4	13.3	3.1	16.3	1.34	2.55
Michalopoulou 2014	OCS+	35.3	9.6	50	9.9	3.2	15	20.1	4.4
	OCS-	37.5	10.9	50	11.2	2.9	10		
Ntouros 2014	OCS+	26.42	6.33	73.7	15.36	4.18	0	9.86	5.43
	OCS-	26.33	4.51	88.9	13.81	3.88	0	0	0
Ongur 2005	OCS+	42.4	10.0	71.6	20.38	1.79	54.5		
	OCS-	43.98	9.18	75,92	14.14	0.88	58.89		
Patel 2010	OCS+	36.0	10.7	83			100	18.0	9.6
	OCS-	37.9	12.7	75			100	2.43	6.8
Sahoo 2018	OCS+	33.47	11.22	57.5	11.97	3.56	65	17.92	5.20
	OCS-	32.87	9.09	41.02	11.20	3.40	69.2	0.87	0.83
Tiryaki 2010	OCS+	33.95	8.47	75			30	14.82	6.0
	OCS-	33.54	9.20	77.3			22.7		
Tonna 2019	OCS+	40.48	12.61	63.0	14.70	5.92	18.5	20.96	9.23
	OCS-	36.88	12.28	73.5	15.00	6.01	8.8	1.44	2.43
Tumkaya 2009	OCS+	33.37	13.37	68.8			0	23.00	8.39
	OCS-	38.63	10.72	56.7			13.3		
Veerman 2016	OCS+	42.82	9.06	63.6	22.55	9.51	100	19.18	9.27
	OCS-	42.22	9.78	78.0	20.61	5.27	100	0.59	1.52
Wang 2019	OCS+	22.00	4.83	63.6			0		
	OCS-	21.50	3.95	60.0			0		
Whitney 2003	OCS+	45.08	8.13	92.3	19.58	5		16.08	6.57
	OCS-	44	9.44	100	18.39	5.7		0.57	1.71
Whitton 2013	All	43.6	9.8	41					

Means and standard deviations (SD) or percentages of confounders per group. OCS+ = people with obsessive-compulsive symptoms. OCS- = people without obsessive-compulsive symptoms. <5 = duration of psychotic illness less than 5 years. >5 = duration of psychotic illness more than 5 years.

8. Meta-regression tables

Below you can find the values entered as covariates into the meta-regression and the statistics for the 19 meta-regressions we conducted. P-values for coefficients lower than 0.05 are marked in bold.

Author & Year	Subgroup	YBOCS OCS+	OCS/OCD	Mean age in years	Mean PANSS positive
Berman 1998		19.9	OCS = 0	50.66	20.87
Bleich-Cohen 2014		20.2	OCD = 1	26.48	
Borkowska 2003		23	OCD = 1	29.75	
Focseneanu 2016		23.11	OCD = 1	23.43	23.93
Frias 2014		18.67	OCD = 1	34.48	16.00
GROUP study		14.64	OCS = 0	27.62	13.92
Hamid 2010			OCD = 1	35.94	
Hermesh 2003		21.5	OCD = 1	32.08	
Hwang 2000		27.9	OCS = 0	37	23.7
Kazhungil 2017		24.4	OCD = 1	28.9	20.56
Kim 2015	<5 years DOI		OCS = 0	28.62	15.89
Kim 2015	>5 years DOI		OCS = 0	36.89	18.05
Kontis 2016		18.09	OCS = 0	42.64	18.49
Kumbhani			OCS = 0	40.69	
Lee 2009		14	OCD = 1	30.41	12.78
Lysaker 2000			OCS = 0	40.02	17.63
Lysaker 2002		22	OCS = 0	42.2	
Lysaker 2004	Good/moderate function	18	OCS = 0	44.62	18.72
Lysaker 2004	Poor function	21	OCS = 0	46.30	18.68
Manheim study		16.5	OCS = 0	37.60	13.58
Michalopoulou 2014		20.1	OCD= 1	36.4	10.55
Ntouros 2014		9.86	OCS= 0	26.38	14.72
Ongur 2005			OCS = 0	43.79	14.88
Patel 2010		18	OCD = 1	37.09	
Sahoo 2018		17.92	OCD = 1	33.17	11.59
Tiryaki 2010		14.82	OCD =1	33.69	
Tonna 2019		20.96	OCS = 0	38.47	14.87
Tumkaya 2009		23	OCD = 1	36.80	
Veerman 2016		19.18	OCS = 0	42.34	21.01
Wang 2019			OCD = 1	21.76	
Whitney 2003		16.08	OCS = 0	44.52	18.96
Whitton 2013			OCS = 0	43.6	

Values entered as covariates into meta-regression. DOI = duration of illness. YBOCS OCS+ = the mean YBOCS score of the comorbid group. OCS/OCD= whether the criterion for the comorbid group was a full diagnosis of OCD or only OCS.

Covariate	N studies	Covariate	Coefficient	SE	95% lower	95% upper	2-sided p-value
**Processing speed**							
Age	12	**Intercept**	0.7278	0.4229	-0.1011	15.568	0.0853
		**Age**	-0.0281	0.0129	-0.0533	-0.0028	**0.0292**
		I^2 = 13.64%					
		R^2 analog = 0.70					
OCD	12	**Intercept**	-0.2363	0.126	-0.4832	0.0105	0.0606
		**OCD**	0.101	0.1885	-0.2683	0.4704	0.5918
		I^2 = 42.33%					
		R^2 analog = 0.00 (computed value is -0.39)					
YBOCS OCS group	10	**Intercept**	-0.6316	0.6552	-19.158	0.6526	0.335
		**YBOCS OCS**	0.0229	0.0343	-0.0445	0.0902	0.5059
		I^2 = 53.74%					
		R^2 analog = 0.00 (computed value is -0.48)					

SE= Standard Error, 95% lower = lower limit of the 95% confidence interval, 95% upper = upper limit of the 95% confidence interval

Covariate	N studies	Covariate	Coefficient	SE	95% lower	95% upper	2-sided p-value
**Immediate visual memory**							
Age	11	**Intercept**	-0.3412	0.9016	-21.083	14.259	0.7051
		**Age**	0.008	0.0228	-0.0367	0.0527	0.7261
		I^2 = 54.55%					
		R^2 analog = 0.00 (computed value is -0.15)					
OCD	11	**Intercept**	-0.0519	0.1629	-0.3713	0.2674	0.7499
		**OCD**	0.0698	0.2787	-0.4765	0.6161	0.8022
		I^2 = 55.62%					
		R^2 analog = 0.00 (computed value is -0.21)					
YBOCS OCS group	11	**Intercept**	-0.6403	0.8607	-23.273	10.467	0.4569
		**YBOCS OCS**	0.0313	0.0436	-0.0542	0.1168	0.4728
		I^2 = 52.81%					
		R^2 analog = 0.00 (computed value is -0.09)					

SE= Standard Error, 95% lower = lower limit of the 95% confidence interval, 95% upper = upper limit of the 95% confidence interval

Covariate	N studies	Covariate	Coefficient	SE	95% lower	95% upper	2-sided p-value
**Working memory**							
		**Intercept**	0.9334	0.455	0.0417	18.252	0.0402
		**Age**	-0.0281	0.013	-0.0536	-0.0026	**0.0308**
Age	15	I^2 = 38.40% R^2 analog = 0.11					
		**Intercept**	-0.1738	0.1123	-0.3938	0.0463	0.1217
		**OCD**	0.3254	0.1703	-0.0083	0.6591	0.056
OCD	15	I^2 = 38.53% R^2 analog = 0.15					
		**Intercept**	0.5322	0.4823	-0.4131	14.776	0.2698
		**PANSS positive**	-0.038	0.0296	-0.0961	0.0201	0.1998
PANSS positive	11	I^2 = 33.06% R^2 analog = 0.00 (computed value is -0.04)					
		**Intercept**	0.3475	0.5847	-0.7985	14.936	0.5523
		**YBOCS OCS**	-0.0188	0.0309	-0.0794	0.0418	0.543
YBOCS OCS group	12	I^2 = 35.59% R^2 analog = 0.00 (computed value is -0.62)					

SE= Standard Error, 95% lower = lower limit of the 95% confidence interval, 95% upper = upper limit of the 95% confidence interval

Covariate	N studies	Covariate	Coefficient	SE	95% lower	95% upper	2-sided p-value
**Set shifting**							
		**Intercept**	-1.107	13.093	-36.733	14.592	0.3978
		**Age**	0.0266	0.0349	-0.0418	0.0949	0.4462
Age	13	I^2 = 81.63% R^2 analog = 0.00 (computed value is -0.40)					
		**Intercept**	-0.0617	0.2154	-0.4839	0.3606	0.7746
		**OCD**	-0.1429	0.3533	-0.8353	0.5495	0.6859
OCD	13	I^2 = 81.60% R^2 analog = 0.00 (computed value is -0.15)					
		**Intercept**	-0.0479	0.869	-1.751	16.553	0.9561
		**YBOCS OCS**	-0.01	0.0437	-0.0957	0.0757	0.8194
YBOCS OCS group	10	I^2 = 74.28% R^2 analog = 0.00 (computed value is -0.36)					

SE= Standard Error, 95% lower = lower limit of the 95% confidence interval, 95% upper = upper limit of the 95% confidence interval

Covariate	N studies	Covariate	Coefficient	SE	95% lower	95% upper	2-sided p-value
**Cognitive inhibition**							
Age	10	**Intercept**	13.205	0.6848	-0.0216	26.627	0.0538
		**Age**	-0.0444	0.0197	-0.0831	-0.0058	**0.0242**
		I^2 = 36.11%					
		R^2 analog = 0.59					
OCD	10	**Intercept**	-0.6025	0.2037	-10.017	-0.2033	0.0031
		**OCD**	0.5867	0.2459	0.1047	10.687	**0.017**
		I^2 = 33.10%					
		R^2 analog = 0.63					

SE= Standard Error, 95% lower = lower limit of the 95% confidence interval, 95% upper = upper limit of the 95% confidence interval

Covariate	N studies	Covariate	Coefficient	SE	95% lower	95% upper	2-sided p-value
**Cognitive flexibility**							
Age	12	**Intercept**	18.825	0.8569	0.2031	3.562	0.028
		**Age**	-0.0577	0.0239	-0.1045	-0.011	**0.0155**
		I^2 = 73.33%					
		R^2 analog = 0.34					
OCD	12	**Intercept**	-0.4826	0.2822	-10.356	0.0705	0.0872
		**OCD**	0.5663	0.3682	-0.1555	1.288	0.1241
		I^2 = 79.58%					
		R^2 analog = 0.02					

SE= Standard Error, 95% lower = lower limit of the 95% confidence interval, 95% upper = upper limit of the 95% confidence interval

Covariate	N studies	Covariate	Coefficient	SE	95% lower	95% upper	2-sided p-value
**Abstract thinking**							
Age	12	**Intercept**	0.5925	0.8073	-0.9897	21.748	0.463
		**Age**	-0.0199	0.0209	-0.0608	0.0211	0.341
		I^2 = 49.10%					
		R^2 analog = 0.05					
OCD	12	**Intercept**	-0.2859	0.1472	-0.5745	0.0027	0.0521
		**OCD**	0.3533	0.2544	-0.1452	0.8518	0.1648
		I^2 = 48.76%					
		R^2 analog = 0.07					

SE= Standard Error, 95% lower = lower limit of the 95% confidence interval, 95% upper = upper limit of the 95% confidence interval

9. Publication bias	Rosenthal's N	Orwin's N	Trim & fill N imputed	Point estimate Trim & fill	Original point estimate	Funnel plot
Attention						
Processing speed	1	28	0	NA	-0.133	Minor bias
*Processing speed: TMTA	0	27	1	-0.22902	-0.177	Minor bias
**Processing speed: not purely RT based	11	27	1	-0.22668	-0.190	Minor bias
**Processing speed: purely RT based	0	10	0	NA	-0.105	Minor bias
Sustained attention	0	7	0	NA	-0.107	Evenly spread
Memory						
Working memory	0	NA	0	NA	-0.030	Evenly spread
*Working memory: digit span	0	10	0	NA	-0.097	Evenly spread
Immediate visual memory	0	NA	0	NA	-0.03	Evenly spread
Delayed visual memory	0	1	1	0.02056	0.051	Minor bias
Trial 1 verbal memory	0	7	2	-0.03230	0.224	Minor bias
Sum of trials verbal memory	0	NA	1	0.0495	-0.035	Evenly spread
Delayed verbal memory	0	NA	2	0.06343	0.023	Minor bias
Executive function						
Fluency	0	26	2	-0.31561	-0.123	Minor bias
Cognitive inhibition	3	29	1	-0.26269	-0.208	Some bias
*Cognitive inhibition: Stroop	2	26	1	-0.26269	-0.208	Some bias
Cognitive flexibility	2	38	3	-0.37413	-0.150	Some bias
*Cognitive flexibility: TMTB seconds	0	23	3	-0.48077	-0.152	Some bias
Set shifting	0	NA	2	0.02235	-0.111	Minor bias
*Set shifting: WCST	0	NA	2	0.1353	-0.081	Minor bias
Abstract thinking	1	26	1	-0.23509	-0.168	Some bias
Planning	0	9	1	-0.07998	-0.229	Minor bias
Reasoning	1	20	1	-0.1115	-0.281	Minor bias
Other						
Facial affect recognition	0	1	1	-0.09539	-0.093	Some bias
Visual spatial ability	0	3	2	0.17002	-0.038	Some bias

*Statistics relating to publication bias. For Orwins's N the effect size was set at 0 with a threshold of (-)0.05. When the effect size was already below 0.05 studies are marked NA. Trim and fill shows the number of imputed studies and the funnel plots shows the result of visual inspection. Rows marked with * are subgroup analyses on specific cognitive tests done as sensitivity analyses. Results marked with ** are post-hoc subgroup analyses.*

10. Results not included in meta-analysis

Below you can find a table with an overview of results from cognitive tests that were mentioned in the primary studies that could be grouped into a domain, but that did not occur 4 times and therefor were not included in the meta-analysis.

	Subgroup	N subjects	Cognitive task	Score used	Mean	SD	Correlation coefficient	
**Gambling task**								
Kazhungil 2017	OCS+<br>OCS-	30<br>30	Iowa Gambling Task	IGT difference	2.13<br>7.93	18.66<br>24.99		OCS- performed better
Patel 2010	OCS+<br>OCS-	12<br>16	CANTAB Cambridge Gambling Task	risk taking<br>decision latency<br>risk taking<br>decision latency	0.54<br>4.95<br>0.48<br>4.80	0.17<br>3.41<br>0.17<br>2.86		OCS+ took more risk and longer latency
Whitney 2003	OCS+<br>OCS-	26<br>28	Bechara Gambling Task	advantageous-disadvantageous	-3.46<br>-1.14	24.54<br>28.05		OCS- performed better
**Verbal learning**								
Kim 2015	OCS+<5<br>OCS-<5<br>OCS+>5<br>OCS->5	13<br>53<br>17<br>80	RAVLT	n learning index	6.1<br>4.3<br>5.1<br>4.5	2.7<br>2.4<br>2.7<br>2.4		OCS+ performed better
Tumkaya 2009	OCS+<br>OCS-	16<br>30	RAVLT	learning	10.69<br>10.30	2.82<br>3.06		OCS+ performed better
Focseneanu 2016	OCS+<br>OCS-	17<br>26	RAVLT	learning rate	9.41<br>5.23	7.26<br>4.24		OCS+ performed better

	Subgroup	N subjects	Cognitive task	Score used	Mean	SD	Correlation coefficient	
**Theory of Mind**								
Meijer 2013	YBOCS=0<br>YBOCS=1-7<br>YBOCS=8-15<br>YBOCS=16+	763<br>67<br>100<br>54	Hinting task		18.5<br>18.6<br>18.4<br>18.2	2.1<br>2.1<br>2.1<br>2.1		OCS- slightly better
Whitton 2013		34	Mind in Eyes				-0.17	OCS- performed better
Ntouros 2014	OCS+<br>OCS-	38<br>27	Perception of Social Inference Test	ToM1<br>ToM2<br>ToM1<br>ToM2	22.13<br>24.39<br>24.26<br>27.89	4.69<br>6.09<br>2.14<br>4.37		OCS- performed better
**Verbal recognition**								
Lee 2009	OCS+<br>OCS-	10<br>17	RAVLT	delayed recognition	8.20<br>8.18	2.57<br>3.78		OCS+ performed better
Focseneanu 2016	OCS+<br>OCS-	17<br>26	RAVLT	volume of recognitions correct	12.00<br>11.19	3.33<br>3.26		OCS+ performed better
Veerman 2016	OCS+<br>OCS-	10<br>39	CANTAB VRM	total correct recognition	20.40<br>19.49	3.20<br>3.38		OCS+ performed better
